# Body Composition and Risk of Incident Heart Failure in 1 Million Adults: A Systematic Review and Dose–Response Meta‐Analysis of Prospective Cohort Studies

**DOI:** 10.1161/JAHA.122.029062

**Published:** 2023-06-22

**Authors:** Ayodipupo S. Oguntade, Nazrul Islam, Reem Malouf, Hannah Taylor, Danyao Jin, Sarah Lewington, Ben Lacey

**Affiliations:** ^1^ Clinical Trial Service Unit & Epidemiological Studies Unit (CTSU), Nuffield Department of Population Health (NDPH) University of Oxford UK; ^2^ School of Primary Care, Population Sciences and Medical Education, Faculty of Medicine University of Southampton UK; ^3^ National Perinatal Epidemiological Unit, Nuffield Department of Population Health University of Oxford UK; ^4^ MRC Population Health Research Unit, NDPH University of Oxford UK; ^5^ UKM Medical Molecular Biology Institute (UMBI), Universiti Kebangsaan Malaysia Kuala Lumpur Malaysia

**Keywords:** adiposity, body composition, body mass index, heart failure, systematic review, Obesity, Heart Failure

## Abstract

**Background:**

The aim of this systematic review was to quantify the associations between body composition measures and risk of incident heart failure (HF) and its subtypes in the general population.

**Methods and Results:**

We searched Medline, Embase, and Global Health databases from each database inception to January 19, 2023 for prospective studies reporting on body composition and HF risk. We followed the Preferred Reporting Items for Systematic Reviews and Meta‐Analyses guidelines. The Newcastle‐Ottawa scale was used to assess the risk of bias of included studies. Fixed‐effects models were used for meta‐analysis. Thirty‐five studies were included (n_total_=1 137 044; n_cases_=34 422). Summary relative risk (RR) per 5‐kg/m^2^ higher body mass index was 1.42 (95% CI, 1.40–1.42; 𝜁^2^=0.02, *I* ^2^=94.4%), 1.28 (95% CI, 1.26–1.31; 𝜁^2^=0.01, *I* ^2^=75.8%) per 10‐cm higher waist circumference, and 1.33 (95% CI, 1.28–1.37; 𝜁^2^=0.04, *I* ^2^=94.9%) per 0.1‐unit higher waist–hip ratio. Pooled estimates of the few studies that reported on regional fat suggested significant positive association between HF risk and both visceral fat (RR, 1.08 [95% CI, 1.04–1.12]) and pericardial fat (RR, 1.08 [95% CI, 1.06–1.10]). Among HF subtypes, associations were stronger for HF with preserved ejection fraction than HF with reduced ejection fraction. No study reported on lean mass.

**Conclusions:**

Pooled data suggested strong associations between adiposity and HF. The association with adiposity is stronger for HF with preserved ejection fraction than HF with reduced ejection fraction, indicating that different mechanisms may be at play in etiopathogenesis of HF subtypes. Future studies are needed to investigate role of regional fat mass and lean mass in HF risk.

**Registration Information:**

REGISTRATION: URL: www.crd.york.ac.uk/prospero/. Unique identifier: CRD42020224584.

**Nonstandard Abbreviations and Acronyms**
ARIC: Atherosclerosis in the Community
HFpEF: heart failure with preserved ejection fraction
HFrEF: heart failure with reduced ejection fraction
MESA: Multi‐Ethnic Study of Atherosclerosis
SAT: subcutaneous adipose tissue
VAT: visceral adipose tissue
WC: waist circumference
WHR: waist–hip ratio

**Clinical Perspective**

**What Is New?**

- This large systematic review and meta‐analysis of 34 422 heart failure (HF) events in >1 million individuals provides the most precise estimates to date of the shape and strength of the association of body composition (adiposity and body fat distribution) with incident HF.
- Excess adiposity, visceral fat, and pericardial fat were associated with increased HF risk, with no excess risk at lower adiposity levels, and the body composition measures showed stronger association with HF with preserved ejection fraction than HF with reduced ejection fraction. Overall general adiposity showed stronger association with HF with preserved ejection fraction than central adiposity measures, whereas central adiposity tended to be stronger in HF with reduced ejection fraction than general adiposity.

**What Are the Clinical Implications?**

- Public health guidance for the general population should emphasize weight reduction strategies to reduce the risk of HF even in individuals without cardiovascular disease, and there is a need for further population‐based studies to clarify the role of imaging derived‐body fat distribution over anthropometric adiposity measures in HF and its subtypes in different racial groups.

There are about 65 million new cases of heart failure (HF) globally, and 5‐year mortality following diagnosis continues to exceed 50% in most settings.^1^, ^2^ Higher adiposity has been associated with higher risk of HF. Body mass index (BMI) is the most widely used measure of general adiposity. Although the relationship between HF risk and measures of central adiposity, such as waist circumference (WC) or waist–hip ratio (WHR), has been less well studied than for BMI, central adiposity has been shown in several studies to be a stronger risk factor than general adiposity for HF.^3^, ^4^, ^5^ Furthermore, the value of fat imaging in HF risk evaluation remains unclear and, as yet, no systematic review has investigated its value over conventional anthropometry.

Some studies have shown a linear relationship between HF risk and BMI, whereas others have found a J‐shaped association, with those in the underweight (BMI <18.5 kg/m^2^) and overweight or obese ranges (BMI >25 kg/m^2^) at higher risk than those in the normal range (18.5–25 kg/m^2^).^6^, ^7^, ^8^, ^9^, ^10^, ^11^, ^12^ However, difficulties with accurate assessment of body composition measures in studies that used routine data may have biased the reported associations. Moreover, many studies that reported on specific populations with diseases or studies that did not account for prevalent cardiovascular disease (CVD) at baseline among participants are prone to reverse causality (whereby HF may have affected adiposity) and may have underestimated the strength of observed associations.

HF is a heterogenous condition with different phenotypes of different etiopathogenesis. It has been subtyped using left ventricular ejection fraction obtained from cardiac imaging into HF with preserved ejection fraction (HFpEF), HF with reduced ejection fraction (HFrEF), and, more recently, HF with mildly reduced ejection fraction.^13^, ^14^ Obesity has been shown to be a risk factor for HFpEF in some observational studies, but data on HFrEF are scarce.^3^, ^15^, ^16^, ^17^, ^18^ Furthermore, previous reviews have tended not to assess the effect of body fat distribution on risk of different HF subtypes.^10^, ^12^

In summary, although there is strong evidence of an association of obesity with incident HF, previous systematic reviews may not have sufficiently accounted for reverse causality from prevalent cardiovascular disease in their assessment of the association of HF risk with body composition in the general population. Several prospective studies have been published since the last systematic review in this area, and there is a need for an updated systematic review including all the current evidence.^3^, ^11^, ^19^, ^20^, ^21^, ^22^, ^23^, ^24^, ^25^, ^26^, ^27^, ^28^, ^29^, ^30^ Also, to the best of our knowledge, no meta‐analyses have yet determined the associations between any of the measures of body composition and risk of HF subtypes. This is, in part, because such studies require measures of cardiac function that have not been feasible to include in large‐scale studies until recently. We conducted this systematic review and meta‐analysis of the current evidence from prospective cohort studies conducted in the general population to determine the associations between different measures of body composition and HF risk to address these uncertainties, and to inform efforts to prevent HF. Additionally, we investigated the extent to which the observed associations vary by HF subtypes.

## Review Questions

1. What is the association between adiposity measures (as measured by BMI, WC, and WHR) and HF incidence?
2. What is the association between fat measures (as measured by total body fat, visceral adipose tissue [VAT], subcutaneous abdominal adipose tissue [SAT], and pericardial fat) and HF incidence?
3. To what extent do these associations between body composition and HF incidence vary by age, sex, race, and HF subtypes?

## Methods

The authors declare that all supporting data are available within the article (and its online supplementary files). This systematic review followed the Preferred Reporting Items for Systematic Reviews and Meta‐Analyses guidelines, which is a standardized format for performing systematic reviews.^31^ Previously, we have published the protocol for this review (see S1).^32^ This review was registered on the International Prospective Register of Systematic Reviews‐PROSPERO (number CRD42020224584).^33^ The review involved secondary analyses of previously published studies, and as such, separate institutional review board approval and informed consent were not required. The data that support the findings of this study are available from the corresponding author upon reasonable request.

### Search Strategy

A systematic search of Medline, Embase, and Global Health databases was performed by 2 reviewers (A.S.O. and D.J.) initially from each database inception to December 16, 2020 for articles that reported on the associations between body composition measures and HF risk; the search was updated to include articles published before January19, 2023. The search strategy was developed in conjunction with an information specialist using Medical Subject Headings terms and text words associated with “body composition,” “adiposity,” “lean mass,” “obesity,” “sarcopenia,” “heart failure,” “cardiac dysfunction,” “ventricular dysfunction,” “cardiomyopathies,” “cohort studies,” and “adults.” A reference list of a previous meta‐analysis^12^ and reference lists of relevant studies were also screened for inclusion. The search strategy is shown in Table S1.

### Study Selection and Eligibility Criteria

The titles and abstracts that were retrieved by the electronic searching of databases were imported into Rayyan review manager, a web page portal that allows article screening and selection for systematic reviews, for removing duplicated citations and screening by the reviewers.^34^ Any disagreements about study selection were resolved through discussion between reviewers. Studies were included if they were prospective cohort studies, nested case–control studies, or randomized controlled trials that allowed for determination of the strength of associations between measures of body composition (BMI, WC, WHR, fat mass, lean mass, subcutaneous abdominal fat, and visceral fat) and incident HF risk. Eligible studies were in adults aged ≥18 years and conducted in the general population. Studies were excluded if they were performed in cohorts with specific diseases only (eg, diabetes, hypertension, or coronary heart disease, or if they only recruited individuals with HF at baseline). Studies that did not provide effect sizes of associations between selected body composition measures and HF risk as well as studies with too few HF events (defined as 20 events or fewer) were also excluded. For the meta‐analysis, eligible studies must have reported relative risk (RR) estimates (hazard ratios or risk ratio) with 95% CIs, and for the dose–response analysis, provided quantitative measure of body composition, number of incident cases, or person‐years and noncases.

For cohorts with multiple publications, we included the publication with the largest number of HF events, except where the study with the largest number of events did not provide both categorical and continuous effect sizes for inclusion in both linear and nonlinear dose response analyses. In such cases, the article that reported both categorical and continuous effect sizes was included. Thus, each cohort was only represented once in the meta‐analysis of each body composition measure.

### Data Extraction

A predesigned data extraction form was used to extract the following data from each included publication: first author's last name, publication year, country of study, name of cohort, year of baseline survey, selection criteria for study participants, baseline characteristics (number of participants, mean age, percent men and women), body composition measures investigated (and mean or median, categories of each body composition where available), mean or median follow‐up (years), number of incident events, HF subtype, shape of association, details of statistical analyses (including type of regression models, variables adjusted for, crude and adjusted RRs and 95% CIs), and main study findings.

### Assessment of Bias

Risk of bias was assessed using the Newcastle‐Ottawa Scale.^35^ The Newcastle‐Ottawa Scale uses 3 quality parameters (study selection, group comparability, and outcome assessment), which are divided into an 8‐item list using a point‐score system. The variables in the study selection domain were study representativeness (general adult population), detailed description of participants' selection and eligibility, use of standardized method for measuring body composition, and absence of HF at baseline. Group comparability domain included adjustment for key confounders. Outcome assessment domain included outcome ascertainment by record linkage or adjudication method, follow‐up period >5 years (to allow assessment of reverse causality), and adequacy of follow‐up (complete follow‐up or <10% lost to follow‐up or nondifferential loss to follow‐up). We assigned 1 point for each of the items if the criteria were met except for the item on adjustment for key confounders, which had a maximum score of 2 points (1 point if a study was adjusted for age and sex and an extra point if a study was adjusted for additional confounders).^36^, ^37^ Studies were rated as high quality if they had at least 7 points, whereas studies with <7 points had a high risk of bias.^36^, ^37^ Full details of risk of bias score of all included studies are shown in Table S2.

### Statistical Analysis

For the dose–response meta‐analyses, summary RR (95% CI) per 5‐kg/m^2^ higher BMI, 10‐cm higher WC, 0.1‐unit higher WHR, 1‐unit higher body fat percent, 10‐cm^3^ higher pericardial fat, and 100‐cm^3^ higher abdominal fat were calculated using fixed‐effects models. For each study, the risk estimate from the most fully adjusted model was used, except for when such a model adjusted for additional intermediate factors (eg, hypertension, blood pressure, diabetes). In such cases, the multivariable model without such adjustment was used. The average of the natural logarithm of the RR was calculated using the inverse variance weighting method.^38^ In cases where studies provided RR (95% CI) per unit higher body composition measure, these estimates were scaled to the desired units by exponentiating the RR (95% CI) to the power of desired units. When studies reported RR separately for different subgroups (eg, age, sex, or race) instead of overall summary estimate, the subgroup estimates were combined using fixed‐effects models to obtain an overall summary estimate. Each study was therefore only represented once in each main meta‐analysis, but such subgroup‐specific estimates are presented separately in subgroup analyses.

For the dose–response analysis, where studies reported estimates for categories of body composition measure or subgroups, such estimates were log‐transformed and used to calculate study specific slopes and 95% CIs across categories of body composition measures as described by Greenland and Longnecker to generate overall study‐specific RRs using the glst command in Stata.^39^, ^40^, ^41^, ^42^ This method requires at least 3 categories of the categorical variable, and number of cases and noncases (or person‐time) must not be missing in each category.^42^ Where studies only reported total cases and controls (or person‐years), the total numbers were divided evenly across the categories.^42^ The mean or median of each category of each body composition measure was assigned to the corresponding RR for that category. For studies that did not report the mean or median body composition measure in each category, the midpoint of the range of such category was used as the mean. A comparison of observed and predicted means of adiposity categories in relevant studies is shown in Table S3. When the lowest or highest category was open ended, the width of the interval was assumed to be the same as that of the adjacent category.^42^ Nonlinear dose–response relationship between each body composition measure and HF was determined using the glst package in Stata by fitting restricted cubic splines with 4 knots at 5th, 35th, 65th, and 95th of each body composition distribution.^43^ A likelihood ratio test was used to test nonlinearity by assessing the difference between the linear and nonlinear models.

Heterogeneity between studies was determined using a *Q* test, whereas between‐study variance was assessed using 𝜁^2^ as described by Islas and Rice.^44^ The *I* ^2^ statistics was used to denote the percentage of total variability due to between‐study heterogeneity. To investigate potential sources of heterogeneity, subgroup analyses were done based on sex, age group, race, study region, duration of follow‐up, measured or self‐reported body composition, study quality, and exclusion of CVD at baseline, and adjustment for confounders and potential intermediate factors. Few studies provided estimates for HF subtypes, but where available, these were presented in the meta‐analyses.

To assess the robustness of the overall estimates, sensitivity analyses were done by removing 1 study at a time to determine whether results were influenced by large studies or studies with extreme results. We also assessed whether results were sensitive to quality of studies, estimation of data from presented results, and heterogeneity of study populations by excluding studies with poor‐quality data and studies for which summary data were estimated. Publication bias and small‐study effects were examined by inspecting funnel plots for asymmetry and with the Egger test. The trim and fill method of Duval and Tweedie was used when there was evidence of publication bias on statistical testing.^12^, ^45^, ^46^ All analyses were done in Stata/MP 17.0 (StataCorp, College Station, TX).

## Results

A total of 22 884 records were identified from the initial literature search. After removal of duplicate records (934 records) and title and abstract screening (19 908 records screened), 152 records were assessed for eligibility. We initially identified 35 publications that included 32 prospective cohorts. The updated search on January 19, 2023 yielded an additional 9 publications, including 3 new prospective cohorts. Thus, a total of 44 publications (35 studies) are included in this review (Figure S1).

### Study Characteristics

The review included 35 prospective studies involving 1 137 044 individuals at baseline and 34 422 incident HF cases. There were 20 studies conducted in Europe,^19^, ^24^, ^25^, ^26^, ^29^, ^47^, ^48^, ^49^, ^50^, ^51^, ^52^, ^53^, ^54^, ^55^, ^56^, ^57^, ^58^, ^59^, ^60^ 13 in the United States,^3^, ^5^, ^20^, ^21^, ^22^, ^23^, ^27^, ^28^, ^30^, ^61^, ^62^, ^63^, ^64^, ^65^, ^66^, ^67^ and 2 in Australia.^11^, ^68^ Many of the studies recruited mainly White populations (n=19), 15 studies were multiracial, whereas only the Jackson Heart Study^30^ in the United States recruited only Black participants. Many of the studies recruited middle‐aged or older individuals, with 7 studies recruiting only elderly populations (aged ≥65 years). There were 3 studies that recruited only women, whereas 9 studies recruited only men (Table S4).

The majority of the studies reported on anthropometric measures; 33 studies reported on BMI, 14 studies reported on WC, and 9 studies reported on WHR. Nine studies excluded underweight individuals, whereas only the Australian 45 and Up Study^68^ excluded individuals at extremes of BMI (<15 kg/m^2^ or >50 kg/m^2^). Five publications from 3 studies^3^, ^4^, ^5^, ^69^, ^70^ used imaging methods to measure body fat distribution, whereas 1 study^53^ quantified body fat using bioimpedance. Importantly, no study reported on lean mass.

Studies' outcomes were reported as either first‐ever incident HF events (n=18), or HF hospitalizations (n=8) or composite of hospitalizations or death from HF (n=9). HF events were ascertained by electronic record linkage to hospital data or national death registers in 19 cohorts, whereas 15 cohorts adjudicated outcomes using clinical criteria. Six studies provided information on HF left ventricular ejection subtypes.^3^, ^11^, ^22^, ^27^, ^63^, ^69^, ^70^, ^71^, ^72^ Follow‐up ranged from 3.4 to 35 years. Overall, 15 cohorts were graded as low quality, whereas the remainder were high quality (Table S4).

### Shape of Association and Nonlinear Dose Response Analyses

**Figure 1:**
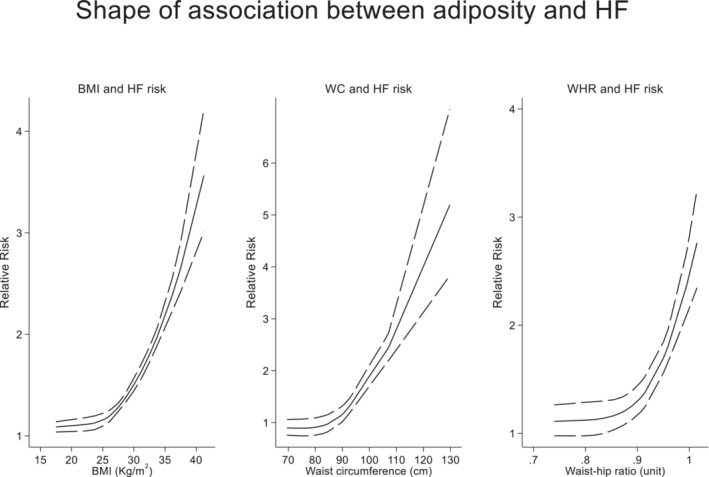
Shape of association between adiposity measures and HF risk. BMI indicates body mass index; HF, heart failure; WC, waist circumference; and WHR, waist–hip ratio.

Most of the studies reported a linear association between BMI and HF risk. However, the Nord‐Trøndelag Health Study 2 (HUNT2)^29^ and Jackson Heart Study^30^ both reported a U‐shaped association, whereas the 45 and Up Study^68^ reported a J‐shaped association between BMI and HF risk. In dose–response analyses, there was a positive curvilinear association between BMI and HF risk (*P*_nonlinearity_<0.001). There was no evidence of excess risk at lower BMI (<25 kg/m^2^), and risk increased approximately linearly above this range. Associations were also curvilinear for both WC and WHR. There was approximate linear increase in risk above a threshold WC of 90 cm and threshold WHR of about 0.9 units (Figure 1). The shape was similar when restricted to studies that excluded CVD at baseline and when restricted to studies with low risk of bias.

### BMI and HF Risk

**Figure 2:**
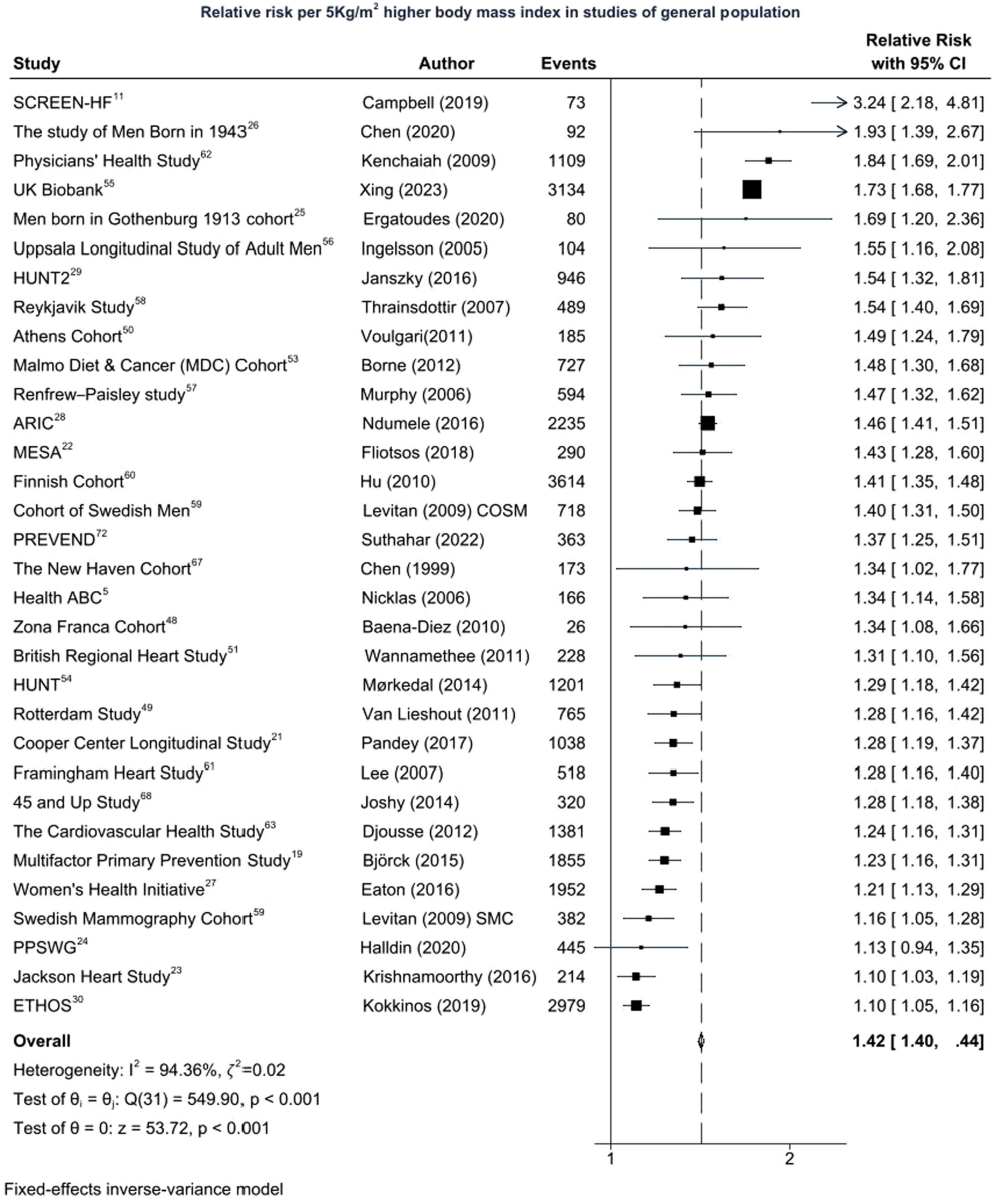
Body mass index and heart failure (HF) incidence: 28 396 HF incident events among 1 095 412 participants. ARIC indicates Atherosclerosis Risk in the Community; ETHOS, Exercise Testing and Health Outcomes Study; Health ABC, Health, Aging and Body Composition Study; HUNT, The Nord‐Trøndelag Health Study; HUNT2, The Nord‐Trøndelag Health Study 2; MESA, Multi‐Ethnic Study of Atherosclerosis; PPSWG, Prospective Population Study of Women in Gothenburg; PREVEND, Prevention of Renal and Vascular End‐Stage Disease; and SCREEN‐HF, Screening Evaluation of the Evolution of New Heart Failure.

Thirty‐two cohorts were included in the dose–response meta‐analysis of the association of BMI and incident HF (Figure 2).^5^, ^11^, ^19^, ^21^, ^22^, ^23^, ^24^, ^25^, ^26^, ^27^, ^28^, ^29^, ^30^, ^48^, ^49^, ^50^, ^51^, ^53^, ^54^, ^56^, ^57^, ^58^, ^59^, ^60^, ^61^, ^62^, ^63^, ^67^, ^68^, ^72^ There were 28 396 HF incident events among 1 095 412 participants. The summary RR of HF for each 5‐kg/m^2^ higher BMI was 1.42 (95% CI, 1.40–1.44). Although there was substantial heterogeneity (𝜁^2^=0.02, *I* ^2^=94.4%, *Q*=549.9, *P*<0.001 for heterogeneity), all studies reported increased risk with higher BMI, but the strength of associations differed between the studies. As shown in the Table, there were significant sex and age differences between studies. Associations were stronger in men (1.29 [95% CI, 1.25–1.32]) than women (1.19 [95% CI, 1.13–1.25]), although studies reporting on men were more heterogeneous than women (𝜁^2^=0.03 and *I* ^2^=93.2% in men versus 𝜁^2^=0 and *I* ^2^=0% in women), but pooled estimate for studies reporting on both sexes (1.48 [95% CI, 1.45–1.50]) were consistent with the overall RR estimates. Associations were also stronger for studies in younger individuals (1.45 [95% CI, 1.39–1.51]; 𝜁^2^=0.02, *I* ^2^=78%) than older individuals (1.30 [95% CI, 1.27–1.33]; 𝜁^2^=0.01, *I* ^2^=80.8%; *P*<0.001). The only study in Black individuals reported a weaker RR of 1.10 (95% CI, 1.03–1.19; 𝜁^2^=0, *I* ^2^=0%; *P*<0.001), whereas multiracial studies reported higher risk than studies in White individuals. There was a temporal increase in strength of associations with longer duration of follow‐up. When studies were stratified based on exclusion of CVD at baseline, there was some evidence of reverse causality. Studies that excluded CVD at baseline reported stronger associations (RR, 1.54 [95% CI, 1.51–1.56]; 𝜁^2^=0.01, *I* ^2^=94.5%) than studies that did not exclude CVD at baseline (RR, 1.28 [95% CI, 1.25–1.30]; 𝜁^2^=0.02, *I* ^2^=87.1%). There was some evidence that the strength of association varied according to adjustment for key confounders (age, sex, education, and smoking), when studies were grouped based on adjustment for these sets of confounders. Studies that adjusted for intermediate factors reported smaller estimates. Further subgroup analyses are shown in Table S5.

**Table 1:** Subgroup Analyses of BMI, Waist Circumference, and Waist–Hip Ratio and Incident HF

Study characteristics	BMI, per 5 kg/m^2^ higher					Waist circumference per 10 cm higher					Waist–hip ratio per 0.1‐unit higher				
	N	RR (95% CI)	*I* ^2^, %	*P*_het_ * value	*P*_het_ † value	N	RR (95% CI)	*I* ^2^, %	*P*_het_ * value	*P*_het_ † value	N	RR (95% CI)	*I* ^2^, %	*P*_het_ * value	*P*_het_ † value
All studies	32	1.42 (1.40–1.44)	94.4	<0.001		14	1.28 (1.26–1.31)	75.8	<0.001		9	1.33 (1.28–1.37)	94.9	<0.001	
Sex															
Women	3	1.19 (1.13–1.25)	0	0.66	<0.001/0.01§	6	1.24 (1.20–1.27)	77.2	0.001	0.003/0.001§	4	1.34 (1.26–1.42)	94.6	<0.001	0.47/0.26§
Men	9	1.29 (1.25–1.32)	93.2	<0.001		8	1.33 (1.29–1.38)	66.1	0.004		4	1.27 (1.20–1.35)	97.3	<0.001	
Men and women	20	1.48 (1.45–1.50)	94.1	<0.001		5	1.27 (1.21–1.33)	12.4	0.34		4	1.32 (1.25–1.40)	71.3	0.02	
Age group															
<65 y	11	1.45 (1.39–1.51)	78.0	<0.001	<0.001/<0.001∥	3	1.37 (1.33–1.42)	0.0	0.94	<0.001/<0.001∥	4	1.48 (1.40–1.56)	96.6	<0.001	<0.001/<0.001∥
≥65 y	13	1.30 (1.27–1.33)	80.8	<0.001		9	1.23 (1.20–1.25)	52.4	0.03		3	1.10 (1.06–1.14)	24.9	0.26	
Unclassified	15	1.43 (1.41–1.46)	96.8	<0.001		6	1.30 (1.24–1.37)	55.9	0.10		6	1.34 (1.27–1.41)	74.2	0.002	
Race and ethnicity															
Black	1	1.10 (1.03–1.19)	0	…	<0.001/0.51¶	0	…	…	…	0.21	0		…	…	<0.001
Hispanic or Chinese	0	…	…	…		0	…	…	…		0		…	…	
White	19	1.36 (1.33–1.39)	65.7	<0.001		9	1.26 (1.22–1.30)	29.9	0.18		7	1.22 (1.18–1.27)	87.0	<0.001	
Multiracial	12	1.47 (1.45–1.50)	97.4	<0.001		5	1.30 (1.26–1.33)	90.2	<0.001		2	1.89 (1.75–2.04)	90.5	0.001	
Region															
Europe	19	1.52 (1.49–1.55)	92.1	<0.001	0.09/<0.001#	9	1.26 (1.22–1.30)	29.9	0.18	0.02/0.35#	7	1.22 (1.18–1.27)	87.0	<0.001	<0.001
United States	11	1.31 (1.29–1.34)	94.3	<0.001		4	1.29 (1.25–1.32)	91.3	<0.001		2	1.89 (1.75–2.04)	90.5	0.001	
Australia	2	132 (1.22–1.43)	95.1	<0.001		1	1.53 (1.34–1.74)	100.0	…		0	…	…	…	
Follow‐up time															
<10 y	11	1.27 (1.23–1.32)	80.8	<0.001	<0.001	7	1.29 (1.24–1.34)	45.6	0.09	0.65	2	1.10 (1.03–1.17)	0.0	0.35	<0.001
≥10 y	12	1.45 (1.43–1.47)	95.5	<0.001		7	1.28 (1.25–1.31)	85.8	<0.001		7	1.42 (1.36–1.47)	94.8	<0.001	
Baseline exclusion of CVD															
Yes	13	1.54 (1.51–1.56)	94.5	<0.001	<0.001	4	1.36 (1.32–1.40)	46.1	0.14	<0.001	3	1.30 (1.22–1.39)	78.7	0.009	0.45
No	19	1.28 (1.25–1.30)	87.1	<0.001		10	1.23 (1.20–1.26)	62.9	0.004		5	1.34 (1.29–1.39)	96.6	<0.001	
HF type															
HFpEF	3	1.42 (1.33–1.51)	85.8	<0.001	<0.001/<0.001**	4	1.29 (1.21–1.37)	72.4	0.01	0.006/0.06**	2	1.35 (1.16–1.58)	0.0	0.49	0.89/0.99^3^
HFrEF	2	1.13 (1.05–1.22)	86.0	0.001		4	1.19 (1.13–1.26)	28.4	0.24		2	1.36 (1.21–1.52)	73.0	0.05	
Unclassified	28	1.41 (1.39–1.43)	93.1	<0.001		11	1.31 (1.28–1.35)	61.3	0.004		7	1.32 (1.28–1.37)	96.2	<0.001	
Adjustment for key confounders only‡															
Yes	7	1.39 (1.35–1.42)	81.1	<0.001	0.02	5	1.29 (1.26–1.33)	89.0	<0.001	0.28	3	1.54 (1.46–1.62)	97.1	<0.001	<0.001
No	25	1.44 (1.41–1.46)	95.3	<0.001		9	1.26 (1.23–1.31)	50.4	0.04		6	1.19 (1.14–1.24)	84.1	<0.001	

N=number of studies in subgroup meta‐analysis (this is not always equal to the total number of studies in the overall analysis). BMI indicates body mass index; CVD, cardiovascular disease; HF, heart failure; HFpEF, heart failure with preserved ejection fraction; HFrEF, heart failure with reduced ejection fraction; and RR, relative risk.

* *P* for heterogeneity within each subgroup.

^†^ *P* for heterogeneity between subgroups.

^‡^ Adequate adjustment is defined as adjusting for at least age, sex, education, and smoking.

^§^ *P* for heterogeneity between men and women (excluding men and women combined).

^∥^ *P* for heterogeneity between age <65 y and age ≥65 y.

^¶^ *P* for heterogeneity between Black and White.

^#^ *P* for heterogeneity between Europe and United States.

** *P* for heterogeneity between HFpEF and HFrEF.

Results were comparable with overall estimates when studies with high risk of bias were excluded from the meta‐analysis (RR, 1.46 [95% CI, 1.44–1.49]; 𝜁^2^=0.02, *I* ^2^=95.6%) and when studies that did not directly report overall RRs were excluded from the meta‐analysis (RR, 1.46 [95% CI, 1.44–1.49]; 𝜁^2^=0.02, *I* ^2^=96.2%) as shown in Figures S2 and S3, respectively. The overall estimate was robust to 'leave one out analysis' and only slightly attenuated when the UK Biobank by Xing et al^55^ was excluded (Figure S4). There was some evidence of publication bias for associations between BMI and HF risk (Egger *P*<0.001) and slight asymmetry of the funnel plot (Figure S5). A trim and fill funnel plot (Figure S5) added 11 studies, and the overall estimate (RR, 1.56 [95% CI, 1.54–1.57]) when these imputed studies were added was stronger than the observed meta‐analysis estimate (RR, 1.42 [95% CI, 1.40–1.44]).

### WC and HF Risk

**Figure 3:**
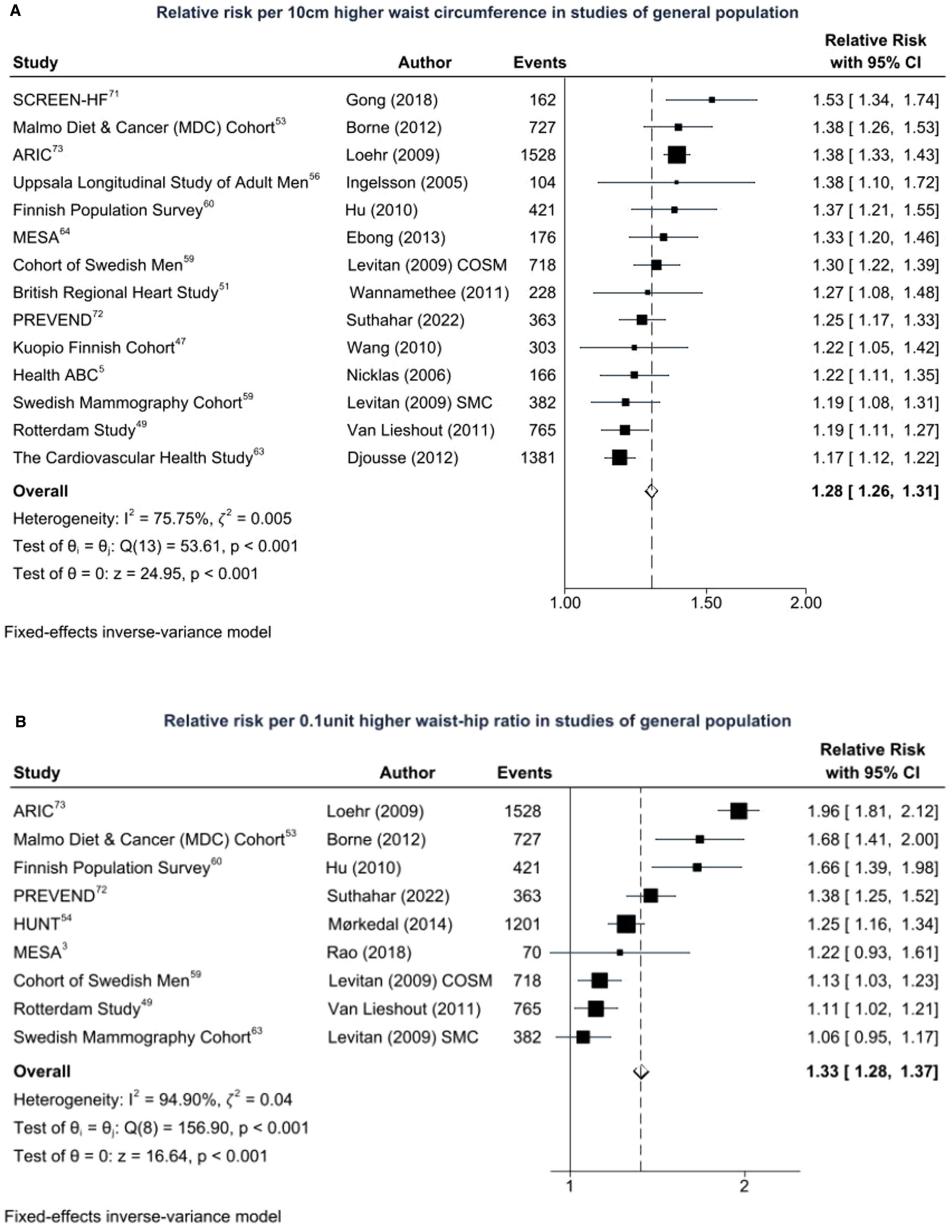
Central adiposity measures and incident heart failure (HF) risk. **A**, Waist circumference and HF incidence: 7424 HF incident events among 219 241 participants. **B**, Waist–hip ratio and HF incidence: 6175 HF incident events among 258 100 participants. ARIC indicates Atherosclerosis Risk in the Community; Health ABC, Health, Aging and Body Composition Study; HUNT, The Nord‐Trøndelag Health Study; PREVEND, Prevention of Renal and Vascular End‐Stage Disease; and SCREEN‐HF, Screening Evaluation of the Evolution of New Heart Failure.

Fourteen cohorts reported the association between WC and incident HF.^5^, ^47^, ^49^, ^51^, ^53^, ^56^, ^59^, ^60^, ^63^, ^64^, ^71^, ^72^, ^73^ There were 7424 HF incident events among 219 241 participants. The summary RR of HF for each 10‐cm higher WC was 1.28 (95% CI, 1.26–1.31). Although there was some heterogeneity (*I* ^2^=75.8%, *Q*=53.6, *P*<0.001 for heterogeneity), the between‐study variance was lower than for BMI (𝜁^2^=0.005), and all studies reported higher risk with higher WC, but the strength of associations differed between the studies (Figure 3A). Associations were stronger in studies of men (1.33 [95% CI, 1.29–1.38]; 𝜁^2^=0.005, *I* ^2^=66.1%) than of women (1.24 [95% CI, 1.20–1.27]; 𝜁^2^=0.004, *I* ^2^=77.2%). Associations were also stronger in younger individuals (1.37 [95% CI, 1.33–1.42]; 𝜁^2^=0, *I* ^2^=0%) than older individuals (1.25 [95% CI, 1.29–1.53]; 𝜁^2^=0.001, *I* ^2^=52.4%). Studies that excluded CVD at baseline reported stronger associations (RR, 1.36 [95% CI, 1.32–1.40]; 𝜁^2^=0.001, *I* ^2^=46.1%) than studies that did not exclude CVD at baseline (RR, 1.23 [95% CI, 1.20–1.26]; 𝜁^2^=0.004, *I* ^2^=62.9%).

When studies were grouped based on adjustment for the set of key confounders (age, sex, education, and smoking), the associations were not material changes, nor did studies differ by adjustment for intermediate factors (Table S5). There was no difference between subgroups based on exclusion of underweight, WC assessment method, and HF ascertainment method (Table S5). Exclusion of studies with a high risk of bias resulted in slight attenuation of the overall estimates (RR, 1.23 [95% CI, 1.20–1.27]; 𝜁^2^=0.01, *I* ^2^=57.3%), whereas exclusion of studies with estimated effect sizes (RR, 1.28 [95% CI, 1.25–1.30]; 𝜁^2^=0.00, *I* ^2^=79.8%) did not (see Figures S6 and S7, respectively). The overall estimate was robust to exclusion of influential studies in leave 1 out analysis (Figure S8). There was no evidence of publication bias for associations between WC and HF risk (Egger *P*=0.75), and no asymmetry of the funnel plot was observed (Figure S9).

### WHR and HF Risk

As shown in Figure 3B, 9 cohorts reported on the association of WHR and incident HF.^3^, ^49^, ^53^, ^54^, ^59^, ^60^, ^72^, ^73^ There were 6175 HF incident events among 258 100 individuals. The summary RR of HF for each 0.1‐unit higher WHR was 1.33 (95% CI, 1.28–1.37). There was substantial heterogeneity, and the between‐studies variance was high (𝜁^2^=0.04, *I* ^2^=94.9%, *Q*=156.9, *P*<0.001 for heterogeneity), but all studies reported higher risk with higher WHR.

As shown in the Table, there was no significant sex difference in association of WHR and HF risk (*P*=0.47). However, there was stronger association in studies of younger individuals (1.48 [95% CI, 1.40–1.56]; 𝜁^2^=0.06, *I* ^2^=96.6%), which were more heterogeneous than older individuals (1.10 [95% CI, 1.06–1.14]; 𝜁^2^=0.001, *I* ^2^=24.9%).

Similar to BMI, there was a temporal increase in strength of associations with longer duration of follow‐up. When studies were grouped based on exclusion of CVD at baseline, there was no evidence of reverse causality (*P*=0.45*)*. There was effect modification based on key confounders and adjustment for intermediate factors (eg, blood pressure accounted for between‐studies variance). Details of other subgroup analyses are shown in Table S5. Unlike BMI and WC, there was no difference in the overall estimates when analyses were restricted to studies with low risk of bias or when studies with estimated effect sizes were excluded (Figures S10 and S11, respectively). The overall estimate was slightly attenuated when the ARIC (Atherosclerosis in the Community) study by Loehr et al^73^ was excluded (Figure S12). There was no evidence of publication bias for associations between WHR and HF risk (Egger *P*=0.28) as shown in Figure S13.

### Body Fat Distribution and HF Risk

**Figure 4:**
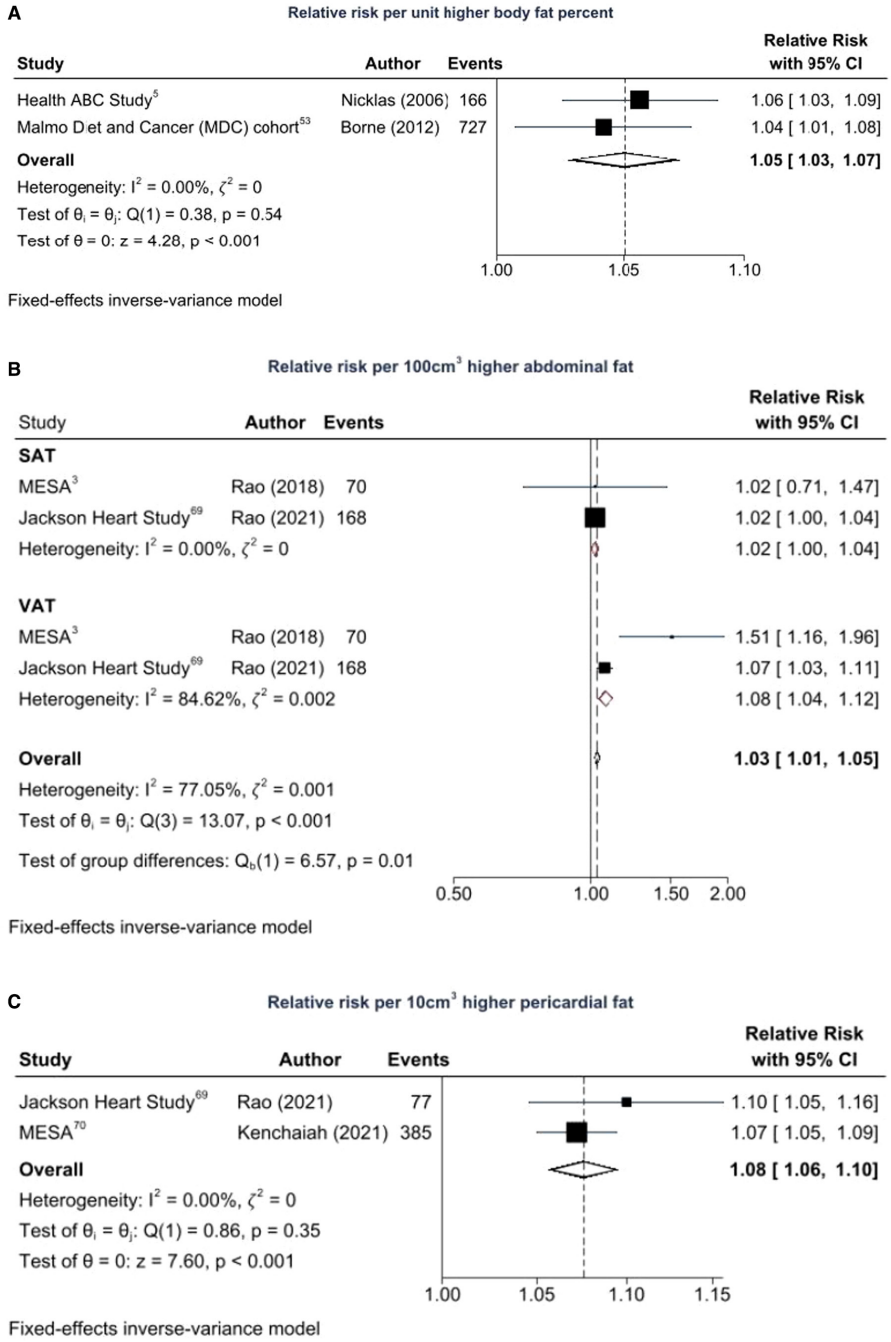
Regional fat measures and incident heart failure (HF) risk. **A**, Body fat percent and HF incidence: 893 HF incident events among 29 088 participants. **B**, Abdominal fat and HF incidence: 238 HF incident events among 4688 participants. **C**, Pericardial fat and HF incidence: 462 HF incident events among 9667 participants. Health ABC indicates Health, Aging and Body Composition Study; MESA, Multi‐Ethnic Study of Atherosclerosis; SAT, subcutaneous abdominal adipose tissue; and VAT, visceral adipose tissue.

Figure 4A through 4C show the association between fat measures and HF risk in the few studies that reported on these measures.^3^, ^5^, ^53^, ^69^, ^70^ Pooled estimates from the Health, Aging and Body Composition (Health ABC)^5^ and Malmö Diet and Cancer (MDC)^53^ cohorts suggested a 5% higher risk of HF per unit higher body fat percent (95% CI, 1.03–1.07) with no difference between studies (𝜁^2^=0, *I* ^2^=0%, *Q*=0.38, *P*=0.54 for heterogeneity). Increased abdominal fat was significantly associated with HF principally due to VAT and not SAT.^3^, ^69^ There was 8% higher HF risk per 100‐cm^3^ higher VAT (95% CI, 1.04–1.12), whereas SAT was not significantly associated with HF incidence (RR per 100 cm^3^, 1.02 [95% CI, 1.04–1.12]). Pooled estimates from the MESA^70^ and Jackson Heart Study^69^ suggested similar 8% higher risk of HF per 10‐cm^3^ higher pericardial fat volume (95% CI, 1.06–1.10), with no difference between studies (𝜁^2^=0, *I* ^2^=0%, *Q*=0.86, *P*=0.35 for heterogeneity).

### Adiposity, Body Fat Distribution, and HF Subtypes

**Figure 5:**
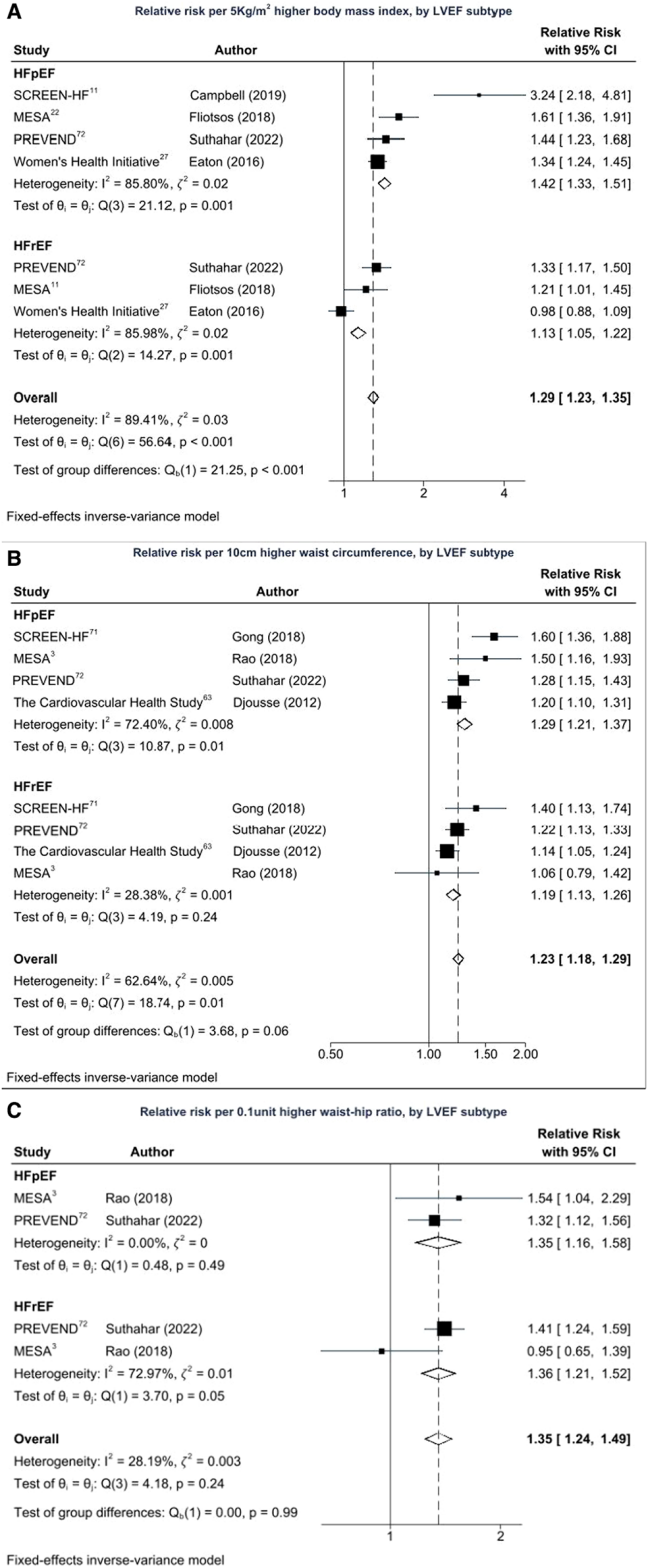
Dose–response meta‐analysis of adiposity measures and incident HF subtypes. **A**, Relative risk per 5‐kg/m^2^ higher body mass index, by left ventricular ejection fraction (LVEF) subtype. **B**, Relative risk per 10‐cm higher waist circumference by LVEF subtype. **C**, Relative risk per 0.1‐unit higher waist–hip ratio by LVEF subtype. HFpEF indicates heart failure with preserved ejection fraction; HFrEF, heart failure with reduced ejection fraction; MESA, Multi‐Ethnic Study of Atherosclerosis; PREVEND, Prevention of Renal and Vascular End‐Stage Disease; and SCREEN‐HF, Screening Evaluation of the Evolution of New Heart Failure.

In analysis restricted to the studies that reported on HF subtypes,^3^, ^11^, ^22^, ^27^, ^63^, ^71^, ^72^ there was a stronger association between BMI and HFpEF (RR, 1.42 [95% CI, 1.33–1.51]; 𝜁^2^=0.02, *I* ^2^=85.8%) than for HFrEF (RR, 1.13 [95% CI, 1.05–1.22]; 𝜁^2^=0.02, *I* ^2^=86.0%) as shown in Figure 5A (*P*<0.001). There was also a trend of stronger association between WC and HFpEF (RR, 1.29 [95% CI, 1.21–1.37]; 𝜁^2^=0.008, *I* ^2^=72.4%) than for HFrEF (RR, 1.13 [95% CI, 1.05–1.23]; 𝜁^2^=0.001, *I* ^2^=28.4%) as shown in Figure 5B (*P*=0.06).^3^, ^63^, ^71^, ^72^ The pooled estimate of the 2 studies that reported on WHR showed no differences between associations of WHR with HF subtypes (*P*=0.99) as shown in Figure 5C.^3^, ^72^ Overall, general adiposity showed stronger association with HFpEF than central adiposity measures, whereas central adiposity tended to be stronger in HFrEF than general adiposity.

**Figure 6:**
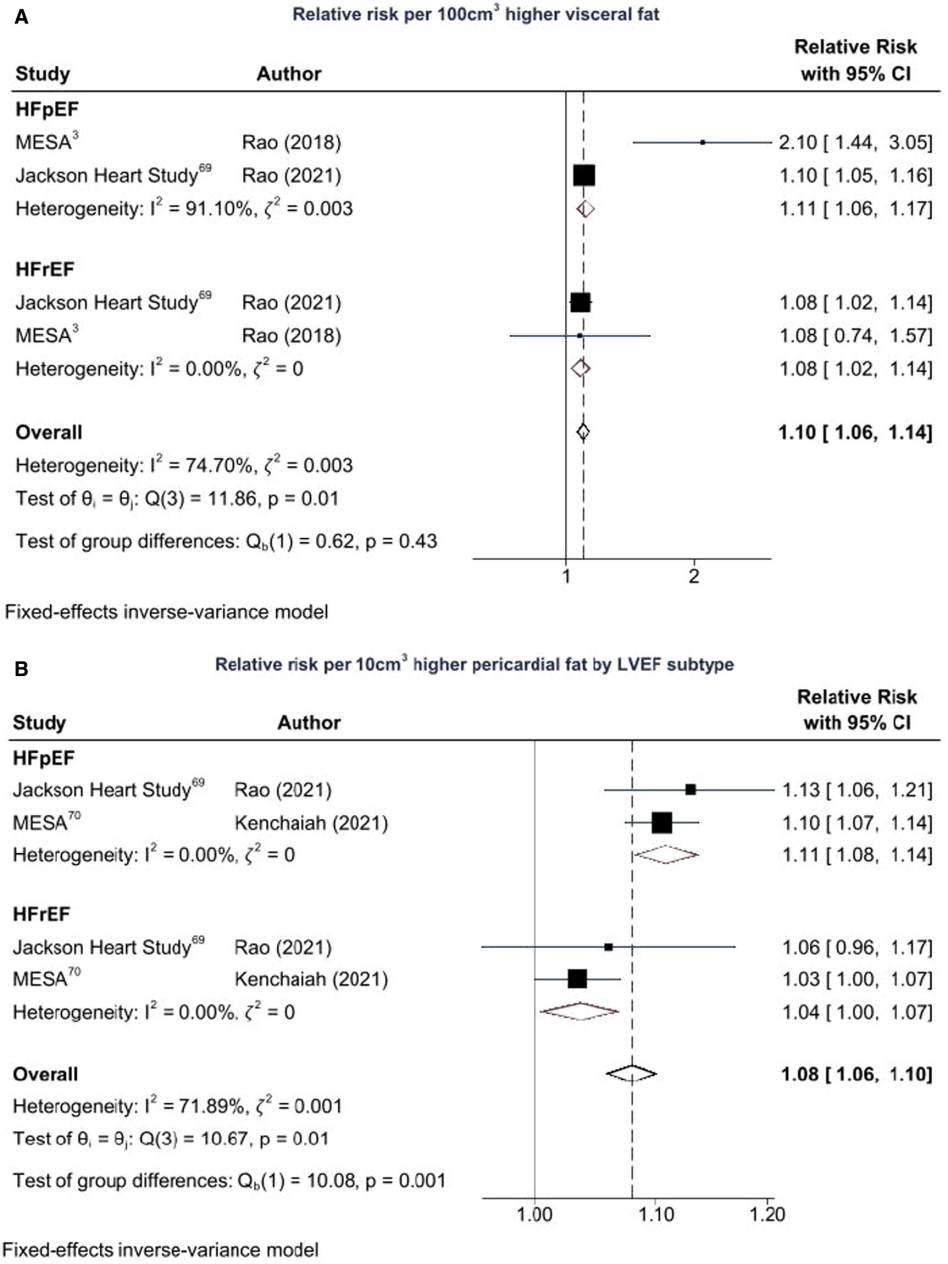
Regional fat and incident heart failure subtypes. **A**, Relative risk per 100‐cm^3^ higher visceral fat by left ventricular ejection fraction (LVEF) subtype. **B**, Relative risk per 10‐cm^3^ higher pericardial fat by LVEF subtype. HFpEF indicates heart failure with preserved ejection fraction; HFrEF, heart failure with reduced ejection fraction; and MESA, Multi‐Ethnic Study of Atherosclerosis.

Figures 6A and 6B show the associations between regional fat and HF subtypes.^3^, ^69^, ^70^ The association between VAT and HF was similar for both HFpEF and HFrEF (*P*=0.43) in the 2 studies that reported on regional fat and HF subtypes. In addition, pericardial fat showed stronger association with HFpEF than HFrEF (*P*<0.001).

## Discussion

Our systematic review is the largest review of prospective cohorts in the general population, with >34 000 HF events in >1 million individuals, and provides the most precise estimates to date of the strength and shape of the associations of body composition measures and incident HF. We have shown that there is an increased risk of HF in association with a range of adiposity and regional fat measures. The reported associations were stronger in men than women for both BMI and WC, but similar in both sexes for WHR. Associations for all adiposity measures were stronger in individuals aged <65 years than older individuals. We have also shown that the risks conferred by different adiposity measures were approximately linear. Above a threshold BMI of 24 kg/m^2^, 90 cm of WC, or 0.9 unit of WHR, HF risk increases log‐linearly. We did not observe excess risk of HF at lower adiposity. In the few studies that reported on total body fat and regional fat, all the fat measures except SAT were associated with higher risk of HF. There was 8% higher HF risk per 100‐cm^3^ higher VAT and per 10‐cm^3^ higher pericardial adipose tissue. We found modest evidence for differences in risk between adiposity and fat measures and HF subtypes. In studies that reported on HF subtypes, we found a stronger positive association for HFpEF than HFrEF. Furthermore, general adiposity was stronger for HFpEF, whereas central adiposity tended to be stronger for HFrEF.

The observed sex differences could be explained by the stronger clustering of cardiometabolic risk factors in men in epidemiologic studies.^74^, ^75^, ^76^, ^77^ Elderly individuals have been shown to be at higher absolute risk of HF in population studies.^78^ However, the relative risk conferred by higher adiposity is probably weaker in them due to weight loss and sarcopenia observed with old age.^79^ It has previously been suggested that lower adiposity could be associated with increased cardiovascular events due to deleterious effects of sarcopenia, poor muscle oxygen uptake, and reduced cardiorespiratory fitness.^80^ However, studies of bariatric surgery in individuals at high risk of HF have shown beneficial effects of weight loss in reducing HF incidence.^80^, ^81^, ^82^ Moreover, studies that have previously reported J‐shaped association reported mainly on HF mortality. Reverse causation and residual confounding from other contributory factors to death, including severe disease, may have been the reason for the observed J‐shaped phenomenon. A previous systematic review by Aune et al^12^ also did not observe a J‐shaped phenomenon for incident HF but rather observed the J‐shaped association with studies reporting on HF mortality.

Few studies reported on HF subtypes, which indicates the likely different mechanisms of the etiogenesis of these HF phenotypes. Increased adiposity has been associated with increased blood volume, higher blood pressure, elevated filling pressures, renin‐angiotensin‐aldosterone system activation, and arterial stiffness, which causes increased left ventricular mass, myocardial concentric remodeling, and hypertrophy, the hallmark of HFpEF.^83^, ^84^, ^85^ Until recently, the role of excess adiposity and body fat distribution in HFrEF was unclear. However, in this meta‐analysis, we have shown that excess adiposity is also associated with higher HFrEF risk in the general population. This could be explained by the depressive effect of lipotoxicity on myocardial fibers and proarrhythmic properties of pericardial adipose tissue. Lipid accumulation in cardiomyocytes and epicardial tissue leads to mitochondrial dysfunction and apoptosis of myocardial cells. This lipotoxicity has been associated with left ventricular remodeling in the transition to HF.^86^, ^87^, ^88^ Accumulation of adipose tissue round the atria and conduction tissue has been linked to increased arrhythmogenesis and atrial fibrillation.^86^, ^88^ There is a 3% to 8% increased risk of atrial fibrillation independent of other cardiovascular risk factors with each unit higher BMI.^83^ Also, VAT, epicardial fat, and vascular tissue secrete proinflammatory cytokines (eg, TNF‐α [tumor necrosis factor‐α], IL‐1 [interleukin‐1], and IL‐6 [interleukin‐6]), which contribute to microvascular endothelial dysfunction and reduced vascular compliance.^89^, ^90^ Reduced vascular compliance and rise in intracardiac pressures lead to further hypertrophy, eccentric remodeling, and eventual myocardial burnout.^85^, ^91^, ^92^ Interestingly, in the Jackson Heart Study, higher BMI was associated with worse peak systolic circumferential strain on cardiac magnetic resonance, which may explain the role of adiposity in HFrEF.^93^ Upregulation of systemic inflammation and blood volume expansion in obesity exacerbate cardiac dysfunction in the failing heart. Myocardial inflammatory changes are also associated with profibrotic signals that contribute to impaired myocardial relaxation and diastolic dysfunction as seen in HFpEF.^83^, ^94^ Although inflammatory biomarkers are elevated in HFpEF, this has not been well described in HFrEF, suggesting that this may not be a prominent pathway in HFrEF.^95^, ^96^ There is a need for more studies to establish the role of adiposity and body fat distribution in HF subtypes, especially HFrEF.

Although there is emerging evidence of the role of lean mass in cardiovascular disease risk, none of the studies in this review reported on lean mass.^97^, ^98^ Sarcopenia has been associated with adverse outcomes in HF, which may point to a possible protective role of muscle mass in HF.^99^, ^100^, ^101^ There is need for studies to investigate the role of muscle mass in HF risk. Moreover, it is still unclear if direct measurement of body fat or its distribution provides extra information above anthropometric measures in HF risk. These are areas of potential investigation for future population cohorts.

This meta‐analysis is not without limitations. There was substantial heterogeneity in the included studies that persisted across different subgroup analyses of the adiposity measures. However, the direction of the observed association was consistent across studies and subgroups. Although 15 cohorts were of low quality, the heterogeneity did not appear to be due to differences in study quality, because the pooled estimates were similar in analyses restricted to studies with low risk of bias. Measurement errors in measurement of anthropometry could potentially explain some of the observed heterogeneity.

There were no studies from Africa and Asia in this meta‐analysis. Body composition might differ between different racial groups and may explain the higher risk of cardiovascular events among South Asian and African individuals compared with European individuals in previous studies.^102^ It was difficult to adequately characterize racial differences in associations between adiposity and HF risk. In the only study that reported on African individuals, the observed associations were weaker than the association seen in White individuals and multiracial studies. There is need for population studies to investigate the racial differences in risk of HF due to adiposity.

Associations were stronger for studies with more events and longer follow‐up, and weaker in studies with shorter follow‐up. Although this could be due to insufficient power of studies with shorter follow‐up to detect incident events, or weight gain over time being responsible for stronger association with time, these explanations do not fully explain this finding. It is well known that body fat distribution is dynamic and shows temporal variation. Studies that use baseline values of anthropometry are prone to underestimate the strength of associations compared with long‐term usual levels of these anthropometric measures. All the included studies used anthropometric measurements at baseline and did not correct for regression dilution bias. There is a need for future studies to explore this phenomenon.

Comparability of studies in meta‐analysis is usually affected by differences in adjustment for confounders and intermediate factors. Several studies either under‐ or overadjusted their reported estimates. Studies that adjusted for intermediate factors and comorbidities reported shallower association. Our sensitivity analyses showed that the heterogeneity is largely explained by differences in handling of covariates and residual confounding.

We may have underestimated or overestimated the effect sizes in the trend estimation for studies that reported categories of anthropometry by assuming that adiposity measures are normally distributed in the general population. This is especially true for studies that did not provide distribution of cases, noncases, and means in each category. However, studies for which effect sizes were estimated had similar association to studies that directly reported effect sizes.

Contrary to our aims, we were unable to extensively investigate the associations between body fat distribution and lean mass with incident HF using imaging methods due to the dearth of studies in this area. Future studies are needed to investigate the independent and additive association of body fat distribution and lean mass in incident HF in the general population.

Our findings have important implications for clinical practice and preventive public health advice for the general population. Both general and central adiposity are associated with increased HF risk, and their routine measurement in the general practice clinics can be used as a predictive marker in individuals at increased risk of HF. We have also shown that VAT and pericardial adipose tissue, but not SAT, are associated with increased HF risk. The stronger association between adiposity and HFpEF than HFrEF points to different roles of adiposity in HF subtypes. Public health guidance for the general population should emphasize weight reduction strategies to reduce the risk of HF even in individuals without CVD. There is a need for larger studies to investigate the role of adiposity in HF subtypes, the added value of fat quantification over anthropometric measures in HF risk prediction, and the racial differences in association between adiposity and HF risk.

## Sources of Funding

This work was funded by a doctoral scholarship to A.S.O. from the Nuffield Department of Population Health, University of Oxford (Oxford, United Kingdom). B.L. acknowledges support from UK Biobank, funded largely by the UK Medical Research Council and Wellcome.

## Disclosures

S.L. reports grants from the Medical Research Council and research funding from the US Centers for Disease Control and Prevention Foundation (with support from Amgen) during the conduct of the study. N.I. reports grants from the Canadian Institutes of Health Research, Office for National Statistics, National Institute for Health and Care Research, and Health Data Research UK unrelated to this study. The remaining authors have no disclosures to report.

## Supporting information

Tables S1–S5
Figures S1–S13
Reference [103]
