# Supplementary material for: Body Composition and Risk of Incident Heart Failure in 1 Million Adults: A Systematic Review and Dose–Response Meta‐Analysis of Prospective Cohort Studies
Source: J Am Heart Assoc. 2023 Jun 22;12(13):e029062. doi: 10.1161/JAHA.122.029062 (PMC10356078; doi:10.1161/JAHA.122.029062)
Supplement: Supplementary file 1 — Tables S1–S5 Figures S1–S13 Reference [103] [file JAH3-12-e029062-s001.pdf]

SUPPLEMENTAL MATERIAL

Table S1. Search strategy for the systematic review.

Search	Keywords	Thesaurus (MeSH)	Textwords
#1	Exposure	Body Composition/<br>Body Weights and Measures/<br>Adipose Tissue/<br>Obesity/<br>Obesity Hypoventilation Syndrome/<br>Obesity, Abdominal/<br>Obesity, Metabolically Benign/<br>Obesity, Morbid/<br>Obesity Management/<br>Bariatrics/<br>Metabolic Syndrome/<br>Adipocytes/<br>Adiposity/<br>Body Fat Distribution/<br>Anthropometry/<br>exp Subcutaneous Fat/<br>exp Subcutaneous Fat, Abdominal/<br>Body Mass Index/<br>Body Weight/<br>Body Height/<br>Waist Circumference/<br>Waist-Height Ratio/<br>Waist-Hip Ratio/<br>Body Constitution/<br>Somatotypes/<br>Body Size/<br>Overweight/<br>Abdominal Fat/<br>Body Weight Changes/<br>Sarcopenia/<br>Thinness/<br>Cachexia/<br>Intra-Abdominal Fat/	((body or abdom* or intraabdom* or central or truncal or trunk or appendicular or subcutaneous or sub-cutaneous or visceral or limb or arm or leg or peripheral or android or gynoid) adj fat?).mp<br>body composition*<br>body weight and measure*<br>adipos*<br>obes*<br>metabolic syndrome*<br>overweight*<br>BMI*<br>adipocyt*<br>fat distribution*<br>fat mass*<br>anthropometr*<br>quetelet* index*<br>body weight*<br>body height*<br>waist circumference*<br>waist-height ratio*<br>hip circumference*<br>waist-hip ratio*<br>body constitution*<br>Somatotypes*<br>body size*<br>body mass*<br>sarcop?enia*<br>thinness*<br>muscle mass*<br>muscle bulk*<br>lean mass*<br>fat-free mass*<br>skeletal bulk*
#2	Outcomes	exp Heart Failure/<br>Pulmonary Edema/<br>Ventricular Dysfunction/<br>Ventricular Dysfunction, Left/<br>Ventricular Dysfunction, Right/<br>exp Cardiomyopathies/<br>Cardiomegaly/<br>Hypertrophy, Left Ventricular/<br>Hypertrophy, Right Ventricular/<br>exp Ventricular Function/	heart failure*<br>cardiac failure*<br>diastolic HF*<br>systolic HF*<br>pulmonary o?dema<br>HFrEF*<br>HFpEF*<br>HFmEF*<br>ventricular failure*<br>biventricular failure*<br>cardiac dysfunction*<br>ventricular dysfunction*<br>cardiomyopath*<br>cardiorenal syndrome*<br>cardiomegaly*<br>ventricular* hypertrophy*<br>cardia* hypertrophy*<br>ventricular function<br>ventricular remodeling*<br>cardia* remodeling*<br>BNP*<br>NT-BNP*<br>natriuretic peptide*
#3	Study type	exp Cohort Studies/<br>Observational Study/	cohort*<br>longitudinal*<br>prospective*<br>follow-up*<br>observational *<br>incidence stud*
#4		#1 AND #2 AND #3	

Table S2. Quality grading of included studies.

S/No	Quality measure (met=1, not met=0)									
	First author (publication year)	Study selection				Group comparability	Outcome assessment			Total quality score (max. 9)
		Representative study[a]	Detailed description of participant selection and eligibility	Standardised or validated method of composition measurement	Absent HF at baseline	Appropriate adjustment[b]	Record linkage or standardised adjudication used for outcome	Follow-up, >5 years	Adequate follow-up (complete follow-up or <10% loss to follow-up)	
1	Chen (1999)[67], USA, The New Haven Cohort	0	1	0	1	2	1	1	0	6
2	He (2001)[66], USA, NHANES 1 Epidemiologic follow-up Study	1	1	1	1	1	1	1	1	8
3	Kenchaiah (2002)[103], USA, Framingham Heart Study	1	1	1	1	1	1	1	1	8
4	Ingelsson (2005)[56], Sweden, The Uppsala Longitudinal Study of Adult Men cohort	1	1	0	1	0	1	1	1	6
5	Nicklas (2006)[5], USA, The Health, Aging and Body Composition study	0	1	1	1	2	1	1	0	7
6	Murphy (2006)[57], Scotland UK, Renfrew–Paisley study	1	1	1	0	2	1	1	0	7
7	Thrainsdottir (2007)[58], Iceland, Reykjavík Study	1	1	0	1	0	1	1	0	5
8	Douglas Lee (2007)[61], USA, Framingham Heart Study	1	1	1	1	1	1	1	0	7
9	Kenchaiah (2009)[62], USA, Physicians' Health	0	1	0	1	2	1	1	0	6
10	Levitan (2009)[59], Sweden<br>Swedish Mammography Cohort	1	1	0	1	2	1	1	0	7
11	Levitan (2009)[59], Sweden, Cohort of Swedish Men	1	1	0	1	2	1	1	0	7
12	Loehr (2009)[73], USA, Atherosclerosis Risk in Communities (ARIC)	1	1	1	1	2	0	1	0	7
13	Hu (2010)[60], Finland,<br>Finnish Population Survey	1	1	1	1	2	1	1	0	8
14	Wang (2010)[47], Finland, Kuopio Finnish Cohort	1	1	1	1	2	0	1	0	7
15	Baena-Diez (2010)[48], Barcelona Spain, Zona Franca Cohort Study	1	1	1	1	1	0	1	0	6
16	Van Lieshout (2011)[49], Netherlands, Rotterdam Study	0	1	1	1	2	1	1	0	6
17	Voulgari (2011)[50], Greece, Athens Cohort	1	1	1	1	2	0	1	0	7
18	Wannamethee (2011)[51], UK, The British Regional Heart Study	0	1	1	1	2	1	1	1	8
19	Djousse (2012)[63], USA, The Cardiovascular Health Study (CHS)	0	1	1	1	2	1	1	0	7
20	Brouwers (2013)[52], Groningen The Netherlands, PREVEND cohort	1	1	0	0	1	1	1	0	5
21	Ebong (2013)[64], USA, Multi-Ethnic Study of Atherosclerosis (MESA)	1	1	1	0	2	1	1	0	7

22	Borne (2014)[53] Ahead of print 2012, Sweden, Malmo Diet and Cancer (MDC) cohort	1	1	1	1	2	1	1	0	8
23	Mørkedal (2014)[54], Norway, HUNT (Nord-Trøndelag Health Study)	1	1	1	1	1	1	1	0	7
24	Joshy (2014)[68], Australia, 45 and Up Study	1	1	0	1	2	1	0	0	6
25	Björck (2015)[19], Gothenburg Sweden, Multifactor Primary Prevention Study	0	1	1	1	2	1	1	1	8
26	Del Gobbo (2015)[20], USA, Cardiovascular Health Study	1	1	1	1	2	1	1	0	8
27	Eaton (2016)[27], USA, Women's Health Initiative	0	1	0	1	2	1	1	0	6
28	Ndumele (2016)[28], USA, Atherosclerosis Risk in Communities (ARIC)	1	1	1	1	2	1	1	0	8
29	Janszky (2016)[29], Norway (HUNT2)	1	1	1	1	2	1	1	0	8
30	Krishnamoorthy (2016)[30], USA, Jackson Heart Study	1	1	1	1	2	1	1	0	8
31	Pandey (2017)[21], USA, Cooper Center Longitudinal Study	1	1	1	0	1	1	1	0	6
32	Rao (2018)[3], USA, Multi-Ethnic Study of Atherosclerosis (MESA)	1	1	1	1	2	1	1	0	8
33	Fliotsos (2018)[22], USA, Multi-Ethnic Study of Atherosclerosis (MESA)	1	1	1	1	2	1	1	0	8
34	Gong (2018)[71], Australia, SCREEN-HF study	0	1	1	1	0	1	0	0	4
35	Pandey (2018)[4], USA, Jackson Heart Study	1	1	1	1	1	1	1	1	8
36	Kokkinos (2019)[23], USA, ETHOS Veteran cohort	0	1	1	1	2	1	1	0	7
37	Kubicki (2020)[65], USA, Southern Community Cohort Study (SCCS)	1	1	0	1	2	1	1	0	7
38	Campbell (2019)[11], Australia, SCREEN-HF	0	1	0	1	0	1	0	0	3
39	Halldin (2020)[24], Gothenburg Sweden, Prospective Population Study of Women in Gothenburg (PPSWG)	0	1	1	1	1	1	1	0	6
40	Ergatoudes (2020)[25], Gothenburg Sweden, Men born in Gothenburg 1913 cohort	0	1	1	1	2	1	1	0	7
41	Chen (2020)[26], Sweden, The Study of men born in 1943	0	1	1	1	2	1	1	0	7
42	Rao (2021)[69] Jackson Heart Study	1	1	1	1	2	1	1	0	8
43	Kenchaiah (2021)[70], MESA	1	1	1	1	2	1	1	1	9
44	Suthahar (2022)[72], The Netherlands, PREVEND	1	1	1	1	1	1	1	0	8
45	Xing (2023)[55], UK, The UK Biobank	1	1	1	1	2	1	1	1	9

[a]Representative cohort defined as general adult population.

[b]Defined as adjustment for at least age and sex (except for studies done in specific sexes or specific age group only). One extra point given for additional adjustment for other lifestyle confounders.

Table S3. Comparison of observed and predicted means of adiposity categories in relevant studies.

Author	Sex	BMI					WC					WHR				
		Categories	Minimum	Maximum	Reported mean	Calculated mean	Categories	Minimum	Maximum	Reported mean	Calculated mean	Categories	Minimum	Maximum	Reported mean	Calculated mean
Kenchaiah (2002)[103]	women	normal	18.5	24.9	22.3	21.7										
	women	overweight	25	29.9	27.1	27.5										
	women	obese	30	34.9	34.1	32.5										
	men	normal	18.5	24.9	23.2	21.7										
	men	overweight	25	29.9	27.2	27.5										
	men	obese	30	34.9	32.7	32.5										
Murphy (2006)[57]	women	normal	18.5	24.9	22.5	21.7										
	women	overweight	25	29.9	27.1	27.5										
	women	obese	30	34.9	33.6	32.5										
	men	normal	18.5	24.9	22.8	21.7										
	men	overweight	25	29.9	27.1	27.5										
	men	obese	30	34.9	32.1	32.5										
	both sexes	normal	18.5	24.9	22.6	21.7										
	both sexes	overweight	25	29.9	27.1	27.5										
	both sexes	obese	30	34.9	33	32.5										
Kenchaiah (2009)[62]	both sexes	lean	18.5	24.9	23	21.7										
	both sexes	overweight	25	29.9	26.6	27.5										
	both sexes	obese	30	34.9	32.4	32.5										
Loehr (2009)[73] white women	white women	normal weight	18.5	25	22.2	21.7	first tertile	74	86.9	78.9	80.45	first tertile	0.79	0.85	0.8	0.82
	white women	overweight	25	29.9	27.2	27.5	second tertile	87	99.9	92.6	93.45	second tertile	0.86	0.92	0.89	0.89
	white women	obese	30	34.9	34.4	32.5	third tertile	100	112.9	111.2	106.45	third tertile	0.93	0.99	0.98	0.96
Loehr (2009)[73] black women	black women	normal weight	18.5	25	22.7	21.7	first tertile	74	86.9	79.4	80.45	first tertile	0.79	0.85	0.8	0.82
	black women	overweight	25	29.9	27.5	27.5	second tertile	87	99.9	93.2	93.45	second tertile	0.86	0.92	0.89	0.89
	black women	obese	30	34.9	35.8	32.5	third tertile	100	112.9	113.4	106.45	third tertile	0.93	0.99	0.98	0.96
Loehr (2009)[73] white men	white men	normal weight	18.5	25	23.1	21.7	first tertile	86.9	94.9	88.9	90.9	first tertile	0.9	0.93	0.91	0.915
	white men	overweight	25	29.9	27.3	27.5	second tertile	95	103	98.4	99	second tertile	0.94	0.97	0.96	0.955
	white men	obese	30	34.9	33	32.5	third tertile	103.1	111.1	110.4	107.1	third tertile	0.98	1.01	1.02	0.995

Loehr (2009)[73] black men	black men	normal weight	18.5	25	22.4	21.7	first tertile	86.9	94.9	86.2	90.9	first tertile	0.9	0.93	0.9	0.915
	black men	overweight	25	29.9	27.4	27.5	second tertile	95	103	98.5	99	second tertile	0.94	0.97	0.96	0.955
	black men	obese	30	34.9	33.6	32.5	third tertile	103.1	111.1	111.8	107.1	third tertile	0.98	1.01	1.02	0.995
Baena-Diez (2010)[48]	both sexes	normal	18.5	25	24.2	21.7										
	both sexes	overweight	25	29.9	28.4	27.5										
	both sexes	obese	30	34.9	33.7	32.5										
Mørkedal (2014)[54]	both sexes	<25 metabolically healthy	20	24.9	22.6	22.45										
	both sexes	<25 metabolically unhealthy	20	24.9	23.9	22.45										
	both sexes	25-<30 metabolically healthy	25	29.9	26.9	27.45										
	both sexes	25-<30 metabolically unhealthy	25	29.9	27.7	27.45										
	both sexes	≥30 metabolically healthy	30	34.9	32.9	32.45										
	both sexes	≥30 metabolically unhealthy	30	34.9	33.3	32.45										
Ndumele (2016)[28]	both sexes	normal	18.5	25		21.7	sex specific WC Quintile 1	52	87	84.5	69.5					
	both sexes	overweight	25	29.9		27.5	sex specific WC Quintile 2	88	94.5	96.8	91.25					
	both sexes	obese	30	34.9		32.5	sex specific WC Quintile 3	95.5	103.5	107.1	99.5					
	both sexes	severely obese	35	39.9		37.5	sex specific WC Quintile 4	104.5	178	122	141.25					
Pandey (2017)[21]	both sexes	normal	18.5	25	22.7	21.7										
	both sexes	overweight	25	29.9	27	27.5										
	both sexes	obese	30	34.9	32.9	32.5										
Fliotsos (2018)[22]	both sexes	normal	18.5	25	22.6	21.7										
	both sexes	overweight	25	29.9	27.4	27.5										
	both sexes	obese	30	34.9	34.5	32.5										
Campbell (2019)[11]	both sexes		18.5	24.9	23.2	21.7										
	both sexes		25	27.4	26.3	26.2										
	both sexes		27.5	29.9	28.7	28.7										
	both sexes		30	32.4	32.7	31.2										

Table S4. Characteristics of studies included in the systematic review.

S/No	First author(Publication year), country, cohort name	Recruitment Year	Study size (% men or women), mean age- years (SD/IQR)	Mean/Median follow-up (years)	Exclusions	Body composition measure	Study outcome	Outcome ascertainment method	Count of incident HF	HF type/aetiologies	Adjustments	Shape of associations	Type of HR	Hazard/Risk Ratio (95%CI)	Study Quality score
1	Chen (1999)[67], USA, The New Haven Cohort	1982	1749 (59% women), 74.2 (6.8) years	7.9 years	Prevalent HF, or ischaemic heart disease	BMI	Incident heart failure	Electronic record linkage and review of hospital records	173	N/A	Age, sex, diabetes, pulse pressure, type of housing	N/A	Per strata	**BMI categories**<br>BMI <24: ref<br>BMI 24-27.9: 1.1 (0.7-1.6)<br>BMI ≥28: 1.6 (1.0-2.4)	6
2	He (2001)[66], USA, NHANES 1 Epidemiologic Follow-up Study	1971-1975	13,643 (59.4% women)<br>Men: 52.2 (15.2) years<br>Women: 48.1 (15.4) years	9 years	Prevalent HF in the 6 months before recruitment, loss to follow-up	BMI	Incident HF	Participants/proxy interviews, review of hospital/nursing home records and death certificates	1382	N/A	Age, sex, race time-dependent history of coronary heart disease	N/A	Overweight (BMI ≥27.3 in women/ ≥27.8 in men) vs normal weight (BMI <27.3 in women/ <27.8 in men)	**BMI categories**<br>Normal weight: ref<br>Overweight women: 1.24 (1.01-1.51)<br>Overweight men: 1.43 (1.19-1.72)<br>Overweight overall: 1.35 (1.17-1.55)	8
3	Kenchaiah (2002)[103], Framingham Heart Study	1976-1979 and 1979-1983)	5881 (54.0% women),	14 years	Under-30 years old, underweight, prevalent HF, missing co-variates, lack of follow-up data	BMI	Incident HF	Adjudication by study panel physicians using Framingham criteria	496	N/A	Age, sex, alcohol, serum total cholesterol, cigarette smoking, valve disease, hypertension, diabetes, electrocardiographic Left ventricular hypertrophy and myocardial infarction	linear	per 1 kg/m² increase of BMI and per strata of BMI	**Per unit higher BMI**<br>Women: 1.07 (1.04-1.07)<br>Men: 1.05 (1.02-1.09)<br>Total: 1.06 (1.04-1.09)<br>**BMI categories**<br>Normal weight (18.5-24.9): ref<br>Overweight (25.0-29.9): 1.34 (1.08-1.67)<br>Obese (≥30.0): 2.04 (1.59-2.63)	8
4	Ingelsson (2005)[56], Sweden, The Uppsala Longitudinal Study of Adult Men cohort	1970-1974	1187 (100% men), ≥70 years	8.9 years (range, 0.01- 11.4 years)	Prevalent HF and valvular disease	BMI	Incident HF hospitalisation	Blinded adjudication of hospital discharge register	104	N/A	Diabetes plus prior acute MI, hypertension, electrocardiographic LVH, smoking, and serum cholesterol level	linear	per SD	BMI 1.35 (1.11-1.65); WC 1.36 (1.10-1.69)	6
5	Nicklas (2006)[5], USA, The Health, Aging and Body Composition study	1997-1998	2435 (56% women),<br>No HF: 74.1 (2.8) years<br>HF: 74.6 (3.0) years	6.1 ± 1.4	Missing last contact date, prevalent adjudicated acute MI, coronary heart disease, heart failure or pacemaker	BMI, WC, waist-thigh ratio (WTR), TFM, BF%, VAT area, SAT area (DXA for fat quantification)	Incident adjudicated chronic HF	Adjudicated HF hospitalisations	166 (54 were diastolic HF)	N/A	age, sex, race, site, education, smoking, and chronic obstructive pulmonary disorder (COPD)	positive	per 4.88 kg/m² increase of BMI, per 7.93% increase of BF%, per 8.76 kg increase of BFM, per 13.38 cm increase of WC, per 0.23 increase of WTR, per 66.37 cm² increase of VAT area, per 124.19 cm² increase of SAT area	BMI: 1.31 (1.13–1.52)<br>WC: 1.33 (1.17–1.50)<br>WTR: 1.19 (1.03–1.38)<br>BF%: 1.55 (1.22–1.96)<br>BFM: 1.31 (1.12–1.54)<br>VAT area: 1.25 (1.08-1.45)<br>SAT area: 1.27 (1.07–1.50)	7
6	Murphy (2006)[57], Scotland UK, Renfrew–Paisley study	1972-1976	15144 (53.8% women), 54(6) years	20	Underweight	BMI	Incident HF	Electronic health linkage	594	N/A	sex, age, adjusted FEV1, number of cigarettes smoked per day and social class.	linear	Per strata	Normal weight: Ref<br>Overweight: 1.26 (1.05-1.50)<br>Obese: 2.09 (1.68-2.59)	7
7	Thrainsdottir (2007)[58], Iceland, Reykjavík Study	1967-1980	7060 (45% women), 33-84 years	13 ± 8 years	diabetes, abnormal glucose regulation or HF at first visit	BMI	Incident HF diagnosis	adjudicated	489	N/A	Sex, IHD, hypertension, cholesterol and smoking	positive	Per 1Kg/m² increase	1.09 (1.07–1.11)	5
8	Douglas Lee (2007)[61], USA, Framingham Heart Study	1968-1994	3362 (57% women), 62 years	20	Prevalent HF, less than 3 BP and BMI measurements in preceding (1970s) and remote (1960s) decades.	BMI	incident HF	Adjudication of medical histories,<br>physical examinations at the heart study, hospitalization records, and<br>communication with personal physicians using the Framingham criteria.	518	N/A	age, sex, serum cholesterol, systolic and diastolic BP, hypertension treatment, diabetes, smoking, valve disease, and previous myocardial infarction (all defined at the baseline examination) and for incidence of an interim myocardial infarction on follow-up.	linear increase	per Kg/m2	Baseline BMI: 1.05 (1.03-1.07)	6
9	Kenchaiah (2009)[62], USA, Physicians' Health	1982	21094 (100% men), 53 years	20.5±5.4 years	Missing height, weight or physical activity at baseline, missing information<br>on other covariates and HF before baseline<br>examination	BMI	incident HF diagnoses	Adjudicated self-reported diagnoses and symptoms	1109	N/A	age,smoking, alcohol, parental history of myocardial infarction, trial group assignment	linear increase	per unit increase and per strata	BMI per unit increase 1.13 (1.11–1.15);<br>**BMI categories:**<br>Lean- reference<br>overweight 1.62 (1.43–1.83)<br>obese 3.38 (2.71–4.21)	5
10	Levitan (2009)[59], Sweden<br>Swedish Mammography Cohort	1997-1998	36873 (100 % women), 48-83 years	7	Prevalent HF, underweight, HF hospitalisation or death in first 2 years of follow-up, absent or incorrect national identification numbers,<br>implausible energy intakes, previous diagnosis of cancer<br>(other than non-melanoma skin cancer)	BMI, WC, WHR, WHtR	incident HF admissions and deaths	Electronic record linkage to the Swedish inpatient and cause of-<br>death registers.	382 women,	N/A	age, education, smoking, alcohol consumption, total physical activity, postmenopausal hormone therapy, living alone, and family history of myocardial infarction.	linear increase	per unit increase in BMI, per 10cm increase in WC, per IQR increase in WHR, per IQR increase in WHtR	BMI: 1.03 (1.01-1.05)<br>WC: 1.19 (1.08-1.31),<br>WHR: 1.05 (0.95-1.15),<br>WHtR: 1.21 (1.06-1.38)	6
11	Levitan (2009)[59], Sweden, Cohort of Swedish Men	1997-1998	43487 (100% men), 45-79 years	7	Prevalent HF, underweight, HF hospitalisation or death in first 2 years of follow-up, absent or incorrect national identification numbers,<br>implausible energy intakes, previous diagnosis of cancer<br>(other than non-melanoma skin cancer)	BMI, WC, WHR, WHtR	incident HF admissions and deaths	Electronic record linkage to the Swedish inpatient and cause of-<br>death registers.	718 men	N/A	age, education, smoking, alcohol consumption, total physical activity, marital status, and family history of myocardial infarction.	linear increase	per unit increase in BMI, per 10cm increase in WC, per IQR increase in WHR, per IQR increase in WHtR	BMI: 1.07 (1.05-1.08)<br>WC: 1.30 (1.21-1.38),<br>WHR: 1.10 (1.03-1.18),<br>WHtR: 1.35 (1.25-1.46)	6
12	Loehr (2009)[73], USA, Atherosclerosis Risk in Communities (ARIC)	1987 and 1989	14641, 54% men with incident HF, 44% men without incident HF),	16	Non-White and non-Black ethnicities, Blacks outside Jackson or Forsyth County, missing anthropometry, prevalent HF, missing criteria to define prevalent HF	BMI, WC	Incident HF (hospitalised and fatal)	Review of participants' interviews, hospital discharges and death certificate files	1528	N/A	age, alcohol use, educational level, smoking status, and center	positive	per SD	**BMI:**<br>Women 1.49 (1.39, 1.59)<br>Men (1.39, 1.57)<br>**WC:**	6

			Incident HF group: 56.8 (5.4) years<br>Non-cases group: 53.8 (5.7) years											Women 1.54 (1.44, 1.66)<br>Men 1.52 (1.43, 1.62)<br>**WHR:**<br>Women 1.59 (1.46, 1.72)<br>Men 1.50 (1.41, 1.60)	
13	Hu (2010)[60], Finland,<br>Finnish Population Survey	Surveys done in 1972, 1977, 1982, 1987,<br>1992, 1997, and 2002.	59178 (51.3% women), 45 (11) years	18.4	Prevalent HF, underweight, incomplete data	BMI, WC, WHR	Incident HF	Electronic record linkage to Finnish Hospital<br>Discharge Register and the National Social Insurance Institution's<br>Register<br>and the Finnish Death Register	3614	N/A	age, study year, education, smoking, alcohol consumption, history of myocardial<br>infarction, valvular heart disease, and diabetes mellitus, systolic blood pressure, total cholesterol, and physical<br>activity.	N/A	Per strata	**BMI:**<br>Men **<25 ref, 25-29.9 1.25 (1.12-1.39) ≥30 1.99 (1.74-2.27)**<br>Women **<25 ref, 25-29.9 1.33 (1.16-1.51) ≥30 1.99 (1.80-2.37)**<br>**WC quartiles:**<br>Men Q1 1.06 (0.69-1.64) Q2 ref Q3 1.21 (0.84-1.76) Q4 1.85 (1.32-2.61)<br>Women Q1 0.48 (0.21-1.13) Q2 ref Q3 1.18 (0.71-1.96) Q4 1.64 (1.02-2.64)<br>**WHR:**<br>Men Q1 0.88 (0.58-1.31) Q2 ref Q3 1.06 (0.74-1.50) Q4 1.71 (1.23-2.37)<br>Women Q1 0.61 (0.31-1.19) Q2 ref Q3 0.98 (0.59-1.63) Q4 1.88 (1.17-3.01)	8
14	Wang (2010)[47], Finland, Kuopio Finnish Cohort	1986-1988	1032, 61.4% women with incident HF, 61.9% women without incident HF),<br>Incident HF group: 69.1 (2.8) years<br>Non-cases group: 68.8 (2.9) years	20	Prevalent HF	WC	Incident HF	Incident HF identified from medical records of the Kuopio University<br>Hospital	303	N/A	age, gender, physical activity during leisure time, smoking, alcohol consumption, antihypertensive medications, total cholesterol and prevalent diabetes	N/A	Per strata	Waist circumference≥94cm (women:≥80 cm): 1.36 (1.03–1.79)<br>Waist circumference≥102cm (women:≥88 cm) 1.40 (1.09–1.80)<br>Waist-to-hip ratio > 0.90 (women: > 0.85) 1.29 (0.94–1.78)<br>BMI≥30 kg/m² 1.55 (1.19–2.02)	7
15	Baena-Diez (2010)[48], Barcelona Spain, Zona Franca Cohort Study	1998	932<br>(BMI <25 61.2% women, BMI 25-29.9 51% women, BMI ≥30 64.4% women), 58 years	9.98	Prevalent HF	BMI	incident HF	Framingham criteria	26 (14 with systolic HF and 12 with non-systolic HF)	analyses presented as any incident HF	age, sex, hypertension, ischaemic heart disease, DM	linear	per unit and per strata	**BMI per unit increase**: 1.06 (1.01–1.10);<br>**BMI categories**<br>BMI <25: reference<br>BMI 25-29.9: 0.79 (0.21–3.00)<br>BMI ≥30:2.45 (1.02–5.61)	8
16	Van Lieshout (2011)[49], Netherlands, Rotterdam Study	1989-1993	5868	10.9 (4.4)	Prevalent HF	BMI, WC, WHR	incident HF	Adjudication of clinical symptoms and signs, hospital discharge letters and notes from general practitioners	765 (373 women, 392 men)	N/A	age, sex, cholesterol, DM, smoking, antihypertensive medications	linear	per SD	BMI 1.20 (1.11 – 1.29)<br>WC 1.21 (1.12 – 1.30)<br>WHR 1.11 (1.02-1.21)<br>Association stronger in men than women; and in middle-aged than elderly	6
17	Voulgari (2011)[50], Greece, Athens Cohort	2003-2005	550	6	Cardiovascular disease, prevalent HF, valvular disease, CKD (eGFR <60ml/min), NSAIDs or corticosteroids in previous 3 months	BMI	Incident HF	Clinical assessment by study physician, LV systolic or diastolic dysfunction by echocardiography	185	N/A	age, sex, impaired glucose tolerance, dyslipidemia, hypertension, current cigarette smoking, physical inactivity, left ventricular hypertrophy and function	N/A	per strata	Normal No MetS Ref(1.00); Normal+MetS 2.33(1.25–4.36) Overweight NoMetS 1.12(0.35–1.33) Overweight+MetS 2.66 (1.73–4.13) Obese No MetS 0.41(0.10–1.31) Obese+MetS 2.13(1.29–3.17)	8
18	Wannamethee (2011)[51], UK, The British Regional Heart Study	1998 -2000	4080 (720 pre-existing CHD 3360 no pre-existing CHD), 100% men, 60-79 years	9	Prevalent HF, underweight, missing BMI	BMI, WC	incident HF	Doctor confirmed<br>diagnosis of HF from primary care records via electronic record linkage.	228 (80 CHD associated HF, 148 non CHD associated HF)	CHD associated HF, non CHD associated HF	age, smoking, physical activity, social class, antihypertensive treatment, prevalent diabetes, prevalent stroke, left ventricular hypertrophy, atrial fibrillation, use of beta-blockers, and FEV1	linear	per unit, per SD and per strata	**Men without CHD:**<br>BMI per SD: 1.19 (1.04–1.37)<br>BMI per unit 1.05 (1.01–1.10)<br>WC per SD 1.17 (0.99–1.37)<br>WC per unit 1.02 (0.99–1.03)<br>**Men with pre-existing CHD:**<br>BMI per SD: 1.32 (1.04–1.66)<br>BMI per unit 1.07 (1.01–1.14)<br>WC per SD 1.30 (1.06–1.59)<br>WC per unit 1.03 (1.00–1.05)	8
19	Djousse (2012)[63], USA, The Cardiovascular Health Study (CHS)	1989-1990; 1992-1993	4861 (42.5% men), mean age in men 73.0 (5.6) years, women (72.3 (5.4) years	11.3	Prevalent HF, missing BMI or WC, moderate/severe<br>aortic or mitral regurgitation or stenosis on echocardiography, missing covariates	BMI, WC,	incident HF	Adjudication and review of self-reported physician diagnosed HF	1381	all HF, HFpEF, HFrEF	age, gender, clinic site, ethnicity,education, alcohol, smoking, physical activity, eGFR, vavular disease, atrial fibrillation, aspirin use, oestrogen use (for women)	linear	per SD	**WC:**<br>all 1.23 (1.16–1.30)<br>men 1.31 (1.19–1.45)<br>women 1.20 (1.11–1.29)<br>HFrEF 1.19 (1.07–1.33),<br>HFpEF 1.27 (1.13–1.42)<br>**BMI**<br>all 1.22 (1.15–1.29),<br>men 1.28 (1.16–1.42),<br>women 1.19 (1.11–1.28)<br>**WHR**<br>All 1.89 (1.03-3.46)- additionally adjusted for BMI	7
20	Brouwers (2013)[52], Groningen The Netherlands, PREVEND cohort	1997-1998	8592<br>(28-75 years old)	11.5 (range 10.8– 11.9)	Insulin dependent<br>diabetes mellitus, pregnant women, and subjects unable<br>or unwilling to participate	BMI used to define obesity as 30Kg/m2	New onset HF	Adjudication of clinical symptoms and signs, hospital records and echocardiographic records	374 subjects. 125 (34%) were classified as HFpEF and 241 (66%) as HFrEF	HFpEF, HFrEF	age and sex	not given	per strata (obesity vs no obesity)	**All HF** 1.93 (1.37–2.73)<br>HRs of HFpEF, HFrEF not reported	6

21	Ebong (2013)[64], USA, Multi-Ethnic Study of Atherosclerosis (MESA)	2000-2002	6809, 45-84 years old	7.6	Participants without baseline measurements of obesity, and<br>those for whom no follow-up was completed	BMI, WC	HF hospitalisations, HF deaths or outpatient diagnosis of HF	Adjudication of hospital records	176	N/A	age, ethnicity, educational<br>status, cigarette smoking, intentional exercise and center.	linear	Per SD	**BMI**<br>Men: 1.33 (1.10-1.61)<br>Women: 1.70 (1.33-2.17)<br>**WC**<br>Men: 1.38 (1.18-1.62)<br>Women: 1.64 (1.29-2.08)	
22	Borne (2014)[53]<br>Ahead of print 2012, Sweden, Malmo Diet and Cancer (MDC) cohort	March 1991 to September 1996	26653 (61.6% women), 45-73 years old	14	history of cardiovascular events<br>(MI or stroke), or HF before baseline exam, missing values of anthropometric measurements and covariates	BMI, WC, WHR, BF%	incident HF hospitalisation as primary diagnosis	Electronic record linkage to Swedish Hospital Discharge Register	727 individuals (398 men and 329 women)	N/A	age, sex, civil status, education level, immigrant status, smoking habits, alcohol consumption, physical activities, blood pressure-lowering medication, lipid-lowering medication, systolic blood pressure, leucocyte count and diabetes mellitus	N/A	per strata	**BMI quintiles**<br>Q1 ref(1.00)<br>Q2 0.98 (0.76–1.25)<br>Q3 1.12 (0.88–1.42)<br>Q4 1.80 (1.45–2.24)<br>**WC quintiles**<br>Q1 1.00 (ref)<br>Q2 0.92 (0.71–1.19)<br>Q3 1.15 (0.90–1.46)<br>Q4 1.87 (1.50–2.34)<br>**WHR quintiles**<br>Q1 1.00 (ref)<br>Q2 1.04 (0.82–1.32)<br>Q3 1.13 (0.90–1.42)<br>Q4 1.77 (1.43–2.19)<br>**BF% quintiles**<br>Q1 1.00(ref)<br>Q2 0.98 (0.77–1.24)<br>Q3 1.18 (0.95–1.47)<br>Q4 1.35 (1.09–1.68)	7
23	Mørkedal (2014)[54], Norway, HUNT (Nord-Trøndelag Health Study)	August 1995 to June 1997	61299 (53.9% women), ≥20 years	12.3	missing information<br>on BMI and individuals with a history of AMI, HF or cerebral stroke at baseline	BMI	first HF hospitalisation	Electronic record to medical records and national death registry. HF was diagnosed by cardiologists using European Society of Cardiology guidelines	1201	N/A	age and sex	N/A	per strata	**BMI categories**<br>BMI <25 metabolically healthy reference<br>BMI <25 metabolically unhealthy 1.3(0.9–1.8)<br>BMI metabolically healthy 25-29.9 1.0(0.8–1.2)<br>BMI 25-29.9 metabolically unhealthy 1.2(1.0–1.4)<br>BMI >30 metabolically healthy 1.6(1.3–2.0)<br>BMI >30 metabolically unhealthy 1.7(1.4–2.0)	8
24	Joshy (2014)[68], Australia, 45 and Up Study	1 January 2006 to 31 December 2008	158546, mean age 57.8 (13.9) years	3.4	invalid age and/or date of<br>recruitment, extreme measures of BMI<br>(<15 kg/m² or >50 kg/m²), cancer or CVD at baseline	BMI	HF hospitalisation	Electronic record linkage	320	N/A	age, sex, region of residence, household income, education, smoking, alcohol intake and<br>health insurance.	J-shape	Per strata	**BMI categories**<br>15-19.99: 1.72 (1.09-2.72)<br>20.0-22.49: Ref<br>22.5-24.99: 0.91 (0.62-1.32)<br>25.0-27.49: 1.13 (0.78-1.64)<br>27.5-29.99: 0.96 (0.63-1.47)<br>30.0-32.49: 1.63 (1.06-2.51)<br>32.5-50: 3.52 (2.39-5.19)	6
25	Björck (2015)[19], Gothenburg Sweden, Multifactor Primary Prevention<br>Study	1970	7495 (100% men), 51.1 (2.3) years	35	Prevalent HF	BMI	Primary or secondary diagnosis of HF	Electronic record linkage to Swedish national Inpatient Register (IPR) and the Swedish<br>Cause of Death register	1855 total, 851 non ischaemic, 1004 ischaemic	any HF, non-ischaemic HF, ischaemic HF	age, IHD, smoking, physical activity and occupational status	linear	per strata	**BMI categories**<br>BMI <22.5 1.00 (ref)<br>BMI 22.5-24.99 1.19 (1.02-1.39)<br>BMI 25-27.49 1.30 (1.11-1.52)<br>BMI 27.5-29.9 1.51 (1.28-1.79)<br>BMI ≥30 1.61 (1.32-1.96)<br>Risk higher in ischaemic than non-ischaemic HF	8
26	Del Gobbo (2015)[20], USA, Cardiovascular Health Study	1989-1990, also recruited 687 African Americans in 1992	4490 (61% women) mean age 72 years	21.5	prevalent HF or moderate and/or severe mitral or aortic regurgitation at baseline, missing<br>information on lifestyle risk factors, or implausible energy intake	BMI and WC	incident HF	Adjudication of outpatient and inpatient medical<br>records, diagnostic tests, clinical consultations, and<br>interviews	1380 (336 in obese, 1044 in non-obese)	N/A	age, sex, race, enrolment site, education, annual income	N/A	per strata	**BMI categories**<br>BMI ≥30: reference,<br>BMI 30: 0.66 (0.62 to 0.82)<br>**WC categories**<br>WC < 88cm women, < 92cm men: 0.76 (0.68-0.86)<br>WC ≥ 88cm women, ≥ 92cm men: 1.0 (ref)	7
27	Eaton (2016)[27], USA, Women's Health Initiative	1993-1998	42170 (100% women), 50-79 years old	13.2	Self-reported prevalent HF, chronic HF on first adjudication, self-reported race: Asian/Pacific islander, Native American, or unknown<br>race/ethnicity	BMI	hospitalised HF	Adjudication of self-reported HF hospitalisation	1952 in total, 902(46.2%) HFpEF, 508 (26.0%) HFrEF, 533 (27.3%) HF unknown ejection fraction, and 9 HF with recovered HF	HFpEF and HFrEF	age, education, family income, history of MI, history of CHD, stroke ever, hypertension, treated diabetes mellitus, history of cancer, hysterecetomy, oophorectomy, atrial fibrillation, chronic lung disease, anemia, comorbidity index, diuretic use, beta blocker use, aspirin use, current hormone therapy, any insurance, alcohol intake, total energy expenditure/week from physical activity, age at screening and heart rate	N/A	per strata	**HFpEF**<br>BMI <25 reference<br>BMI 25-<30 1.11 (0.88–1.40)<br>BMI 30-<35 1.35 (1.06–1.72)<br>BMI ≥35 2.36 (1.84–3.03)<br>**HFrEF**<br>BMI <25 reference,<br>BMI 25-<30 0.91 (0.68–1.21)<br>BMI 30-<35 1.00 (0.74–1.36)<br>BMI ≥35 0.87 (0.61–1.24)	5

28	Ndumele (2016)[28], USA, Atherosclerosis Risk in Communities (ARIC)	1987 to 1989	13730	23	Prevalent HF or cardiovascular disease, missing BMI, underweight (BMI <18.5Kg/m^2), not of either black or white race	BMI and WC	incident first hospitalization or death related to HF	Adjudication of discharge codes from hospitalizations<br>and death certificates	2235	N/A	age, race, sex, alcohol use, smoking status, physical activity, occupation, and education level	linear increase	per strata	**BMI categories**<br>normal weight Reference; overweight 1.38 (1.23–1.54);<br>obese 2.10 (1.85–2.38);<br>severely obese 3.74 (3.24–4.31)<br>**Sex-specific WC quartiles**<br>Q1 1.0 (Ref);<br>Q2 1.43 (1.23–1.67);<br>Q3 1.74 (1.50–2.00)<br>Q4 3.01 (2.63–3.44)	8
29	Janszky (2016)[29], Norway (HUNT2)	August 1995 to June 1997	26097 (56.3% women), 61.0 (12.2) years	11.4	Underweight BMI, missing information on BMI and individuals with a history of AMI, HF or stroke at baseline.	BMI	incident HF	Electronic record linkage to the two hospitals of Nord-Trøndelag County	946	N/A	sex, age, smoking status, level of education, marital status, physical activity, and alcohol consumption	U shaped for average BMI and HF risk	per strata	BMI <24.9 Reference<br>BMI 25.0-27.4: 1.07 (0.84-1.36)<br>BMI 27.5-29.9: 1.26 (0.98-1.61)<br>BMI 30.0-32.4: 1.26 (0.93-1.70)<br>BMI 32.5-34.9: 1.84 (1.30-2.59)<br>BMI ≥35: 2.65 (1.86-3.77)	8
30	Krishnamoorthy (2016)[30], USA, Jackson Heart Study	September 2000 and January 2013	5184	7	HF at baseline	BMI categorised into normal (<25 kg/m2), overweight (25 to<30 kg/m2),obese (30 to<35 kg/m2), and morbidly obese (≥35 kg/m2)	HF hospitalisation	Adjudication of HF hospitalisations using modified Gothenburg<br>criteria	214	N/A	age, sex, prior myocardial infarction, hypertension, prior stroke, diabetes mellitus, chronic lung disease, smoking status, systolic blood pressure, pulse, sodium, estimated glomerular filtration rate, haemoglobin, glucose, high-sensitivity C-reactive protein, triglycerides, high-density lipoprotein cholesterol, low-density lipoprotein cholesterol, left ventricular ejection fraction, left ventricular hypertrophy, left ventricular diameter, beta-blocker, angiotensin-converting enzyme inhibitor or angiotensin II receptor blocker, statin, antiplatelet agent, missing medication status and prevalent HF at examination 1	U shaped	per Kg/m2 increase and per strata	**BMI per unit increase**:<br>crude 1.03 (1.01-1.04),<br>adjusted 1.02 (1.01-1.04);<br>**BMI categories (adjusted)**<br>normal 1.00 [Reference]<br>overweight 0.79 (0.54–1.14),<br>obese 0.68 (0.46–1.02),<br>morbidly obese 0.97 (0.66–1.44)	6
31	Pandey (2017)[21], USA, Cooper Center Longitudinal Study	1970-2009	19485, individuals ≥65 years old	6.67	self-reported history of myocardial<br>infarction or stroke at study entry, <65 years of age (due to Medicare coverage for disability, endstage<br>renal disease, and other factors), Individuals lacking both Part A and B Medicare coverage and<br>those with Health Maintenance Organization exclusions	BMI	HF hospitalisation	Electronic record linkage to Medicare heart failure billing codes	1038	N/A	age and sex	linear	per 3Kg/m2	BMI per 3Kg/m2 increase: 1.25 (1.17 to 1.32)	6
32	Rao (2018)[3], USA, Multi-Ethnic Study of Atherosclerosis (MESA)	2002-2004, and between 2004-2005	1806 (48.4% men), 64.5 (9.6) years	10.5	Cardiovascular disease at baseline, HF event before the abdominal CT scan date, missing subcutaneous fat and visceral fat for all slices, missing ejection fraction at time of HF diagnosis, or missing other covariates in main analysis	BMI, WC, WHR, subcutaneous fat, visceral fat	incident HF	Adjudication of medical records	Total HF=70 (34 HFpEF, 36 HFrEF)	HFpEF, HFrEF	age, sex, race/ethnicity, smoking, and physical activity	linear increase	per SD	**All HF**:<br>BMI 1.43 (1.11, 1.84)<br>WC 1.40 (1.08, 1.81)<br>WHR 1.22 (0.93, 1.61)<br>SAT at L2-L3 1.22 (0.92, 1.63)<br>SAT sum of 6 pcs 1.02 (0.73, 1.42)<br>VAT at L2-L3 1.50 (1.16, 1.93)<br>VAT sum of 6 pcs 1.46 (1.15, 1.86)<br>**HFpEF:**<br>BMI 1.73 (1.23–2.42)<br>WC 1.74 (1.23–2.46)<br>WHR 1.54 (1.04–2.30)<br>SAT at L2-L3 1.31 (0.89–1.93)<br>SAT sum of 6 pcs 1.23 (0.79–1.90) VAT at L2-L3 2.06 (1.44–2.95)<br>VAT sum of 6 pcs 1.98 (1.40–2.79)<br>**HFrEF**:<br>BMI 1.14 (0.77–1.68)<br>WC 1.08 (0.74–1.58)<br>WHR 0.95 (0.65–1.39)<br>SAT at L2-L3 1.12 (0.73–1.70)<br>SAT sum of 6 pcs 0.80 (0.47–1.35<br>VAT at L2-L3 1.08 (0.75–1.55)<br>VAT sum of 6 pcs 1.07 (0.76–1.52)	7
33	Fliotsos (2018)[22], USA, Multi-Ethnic Study of Atherosclerosis (MESA)	2000-2002	6437 (47.4% men), 62.2 (10.2) years	13	missing self-reported weight at age 20 or 40 years, had no follow-up information for atherosclerotic cardiovascular disease or HF, or were<br>missing key covariates	baseline BMI	definite or probable HF (hospitalized)	Adjudication of medical records, telephone interviews every 9 to 12 months regarding<br>interim hospital admissions, outpatient cardiovascular diagnoses	290	all HF, HFpEF, HFrEF	adjusted for age at baseline, sex, race/ethnicity, center, and education	curvilinear	per 5units change	**All HF**: 1.43 (1.28, 1.60)<br>**BMI categories**<br>normal: reference<br>overweight 1.24 (0.90, 1.71)<br>obese 1.86 (1.34, 2.60),<br>**HFpEF**: 1.61 (1.36, 1.91)<br>**BMI categories**	8

								and procedures, and deaths;						normal: reference<br>overweight 1.27 (0.77, 2.10)<br>obese 1.862.09 (1.24, 3.52),<br>**HFrEF**: 1.21 (1.01, 1.46)<br>**BMI categories**<br>normal: reference<br>overweight 0.96 (0.59, 1.57<br>obese 1.39 (0.84, 2.30)	
34	Gong (2018)[71], Australia, SCREEN-HF study	May 2007-January 2010	3847 (56.6% men) ≥60 years	4.5	Prevalent HF, LVEF < 50%, significant valve abnormality	Baseline BMI and waist circumference	Incident HF	Adjudication of HF events using ESC criteria of 2012	162 (73 HFpEF, 53 HFrEF, 36 Vavular HF)	All, HFpEF, HFrEF and valvular HF	univariate	N/A	log BMI per doubling<br>per 10cm higher waist circumference	**HFpEF**<br>BMI: 15 (6-35)<br>WC: 1.6 (1.3-1.8)<br>**HFrEF**<br>BMI: 2.0 (0.7-5.4)<br>WC: 1.4 (1.1-1.7)<br>**Valvular HF**<br>BMI: 0.5 (0.1-2.0)<br>WC: 0.9 (0.7-1.2)	4
35	Pandey (2018)[4], USA, Jackson Heart Study	2005-2009 (visit 2)	2602 (35% men), 59 years	7.1	Weight >350pounds, pregnancy/unknown pregnancy status, age <40 years in women or <35 years in men, prevalent HFand loss to follow up	BMI, visceral fat (VAT) and abdominal subcutaneous fat (SAT)	Incident HF	Adjudication of HF events	122	N/A	Age and sex	linear	per strata<br>per SD of VAT and SAT<br>per 1kg/m² BMI	**VAT: 1.29 (1.09-1.52)**<br>Tertile 1: ref<br>Tertile 2: 1.40 (0.84-2.32)<br>Tertile 3: 1.82 (1.14-2.92)<br>**SAT: 1.21 (0.99-1.48)**<br>Tertile 1: ref<br>Tertile 2: 0.81 (0.49-1.32)<br>Tertile 3 1.69 (1.07-2.67)<br>**BMI: 1.05 (1.02-1.08)**	
36	Kokkinos (2019)[23], USA, ETHOS Veteran cohort	veterans who underwent treadmill tes between 1987 and 2017	20 254 (100% men), 58 (11.3) years	mean 3.6 ± 7.7 years, with a median of 13.4 years,	existing HF at the time of exercise testing or developed HF within<br>3months after the exercise test, BMI <18.5Kg/m², unstable or required emergent intervention or were unable to complete the test for orthopaedic, neurologic, or other reasons, exercise<br>capacity <2METs, implanted pacemaker,lost to follow-up or missing data	BMI	Incident HF	Review of VA Computerized<br>Patient Record System (CPRS) using ICD codes for HF	2979	N/A	age, BMI, ethnic origin, beta-blockers, calcium channel block-ers, angiotensin-converting enzyme inhibitors, angiotensin receptor blockers,diuretics, lipid-lowering agents, hypoglycaemic agents, smoking status, type 2 diabetes, dyslipidaemia, and hypertension	linear	per Kg/m2	1.02 (1.01 –1.03)	7
37	Campbell (2019)[11], Australia, SCREEN-HF		3842 (55% men), 70 (65-75) years	Total 5.6 (IQR: 4.5–6.3); HFpEF 4.5 (interquartile range: 2.9–5.5)	known heart<br>failure, ejection fraction <50% or more than mild valve abnormality	BMI, WC	Incident HF (ambulatory and hospital diagnosed)	Adjudication by 2 HF specialists according to European Society of<br>Cardiology (ESC) criteria of 2012	162 (73 with HFpEF, 53 with HFrEF and 36 with VHF)	HFpEF	age, hypertension, diabetes, myocardial infarction, atrial fibrillation, serum amino-terminal pro-B-type natriuretic peptide (NT-proBNP) quintile, haemoglobin, and calcium channel blocker therapy	positive increase across categories	per strata	**BMI**<br>BMI <25 (ref),<br>BMI 25-27.4 : 2.5 (0.9-6.8)<br>BMI 27.5-29.9: 5.4 (2.1-13.8)<br>BMI ≥30: 7.6 (3.3-17.8);<br>**WC quintiles**<br>Q1. 66–94 in men; 57–83 in women: 1(ref)<br>Q2. 95–100 in men; 84–90 in women: 2.8 (0.8-9.7)<br>Q3. 101–105 in men; 91–96 in women: 5.0 (1.6-15.5)<br>Q4. 106–112 in men; 97–104 in women: 4.4 (1.4-13.7)<br>Q5. 113–155 in men; 105–146 in women: 10.2 (3.5-29.6)	6
38	Kubicki (2020)[65], USA, Southern Community Cohort Study (SCCS)	2002-2009	27,078 (62.6% women), 69% black, 54 (47-65) years	5.2 (3.1, 6.7) years	Prevalent HF	BMI	Incident HF	Electronic health record linkage	4341	N/A	Age, sex, race, history of myocardial infarction or coronary artery bypass graft, stroke, transient iscahemic attack, education, annual household income, marital status, enrolment source, diabtes, hypertension, underactivity, smoking, poor diet and serum cholesterol	N/A	BMI ≥25 kg/m² vs normal BMI <25 kg/m²	BMI <25 kg/m²: ref<br>BMI ≥25 kg/m² :1.12 (1.03-1.22)	7
39	Halldin (2020)[24], Gothenburg Sweden, Prospective Population Study of Women in Gothenburg (PPSWG)	PPSWG (1968-1969); PPSWG (1980-1981)	1968-1980 cohort 1132; 1980-1992 cohort 932<br>(100% women)	1968 to 1980 cohort: 44 years; 1980 to 1992: 32 years	previous history, sign or diagnosis of HF	BMI	hospitalisation or mortality for HF	Electronic record linkage to Swedish Hospital Discharge Registry and Swedish National Board of Health and<br>Welfare register of causes of death	1968-1980 cohort 271, 1980-1992 cohort 174	N/A	age	N/A	per strata	**1968-1980 cohort**<br>BMI <25: reference<br>BMI 25- <30: 1.26 (0.90–1.72)<br>BMI ≥30: 1.21 (0.73–1.99)<br>**1980-1992 cohort**<br>BMI <25: reference<br>BMI 25- <30: 1.01 (0.66–1.54)<br>BMI ≥30: 1.27 (0.72–2.21)	6
40	Ergatoudes (2020)[25], Gothenburg Sweden, Men born in Gothenburg 1913 cohort	1963	855 (100% men), 50 years old	21	Not mentioned	BMI	Incident HF hospitalisation or HF death	Electronic record linkage to National Hospital Discharge Register or National Cause of Death Register	80	N/A	hypertension, SBP, smoking, cholesterol, physical activity, alcohol, diabetes, AF and IHD	linear	Per unit BMI	HR per unit: 1.11 (1.04-1.19)<br>Obese vs non-obse BMI: HR 2.25 (1.13-4.51)	6

41	Chen (2020)[26], Sweden, The Study of men born in 1943	1993	798 (100% men),	21	Not mentioned	BMI	HF hospitalisation or HF death or cardiac dysfunction at age 71 years	Electronic record linkage to National Hospital Discharge Register or National Cause of Death Register	92	N/A	smoking, BMI, systolic BP, hyperlipidemia, sedentary lifestyle, and diabetes	linear	Per unit BMI	HR per unit: 1.14 (1.07–1.22)	8
42	Rao (2021)[69], USA, Jackson Heart Study	Exam 2 (2005-2008)	2882 (35% men), 59.4 years	10.6 years	Prevalent HF at exam 2, missing measures for BMI, waist, or hip circumference	Visceral fat (VAT), subcutaneous fat (SAT), pericardial fat (PAT)	All-cause death, HF hospitalisation	Adjudication of HF events	168 HF hospitalisations (in VAT and SAT analyses) and 77 HF hospitalisations in PAT analyses	HFpEF, HFrEF	age, sex, education, and smoking status	linear	VAT- per 100 cm^3<br>SAT- per 100 cm^3<br>PAT- per 10 cm^3	**HF**<br>VAT: 1.07 (1.03–1.11)<br>SAT: 1.02 (1.00–1.04)<br>PAT: 1.10 (1.04–1.15)<br>**HFpEF**<br>VAT: 1.10 (1.04–1.15)<br>PAT: 1.13 (1.06–1.21)<br>**HFrEF**<br>VAT: 1.08 (1.01–1.13)<br>PAT: 1.06 (0.96–1.17)	8
43	Kenchaiah (2021)[70], USA, MESA	July 17, 2000, and August 31, 2002,	6,785 participants (3,584 women and<br>3,201 men), ages 45 to 84	mean: 13.4 (4.6) years; median: 15.7 years; interquartile<br>range: 11.7 to 16.5 years; maximum: 17.5 years	Clinical cardiovascular disease at baseline, no cardiac CT at baseline, participants with suboptimal image quality for pericardial fat volume (PFV) measurement, missing information on newly diagnosed HF during follow-up.	Pericardial fat volume (PFV)	Incident HF	Independent adjudication of HF events	385 participants (5.7%;<br>164 women and 221 men)	HFpEF, HFrEF, HFmEF, HFuEF	age (for every 1-year increase), sex, race (White [referent], Black, Hispanic, Chinese), cigarette smoking (no [referent], past, current), alcohol consumption (no or past [referent],<br>mild-to-moderate, heavy), and vigorous physical activity at baseline	linear	PFV per SD (1 SD = 42 cm^3) higher	**HF**<br>Men: 1.24 (1.12–1.37)<br>Women: 1.68 (1.42–1.98)<br>Both sexes: 1.34 (1.23–1.46)<br>**HFpEF:** 1.52 (1.35–1.72)<br>**HFrEF:** 1.15 (1.00–1.33)<br>**HFmEF:** 1.44 (1.12–1.85)<br>**HFuEF:** 1.23 (0.89–1.70)	9
44	Suthahar (2022)[72], Groningen, The Netherlands, PREVEND	1997–1998	8295 participants (4134 women), 49.8% women, 50 (13) years	11.3 ± 3.1 years	Insuin use, pregnancy, no consent, serious mental illness, life expectancy <1 year, treatment for malignancies (other than non-melanoma sin cancer), HF at baseline, underweight, waist circumference <40cm, missing covariates	BMI, WC, WHR, body shape index (BSI), weight-adjusted-waist index (WWI), body roundness index (BRI) and relative fat mass (RFM)	Incident HF	Independent adjudication of HF events using ESC guidelines	363 incident HF	HFpEF,<br>HFrEF	age, sex	linear	HRs per SD higher adiposity measures	**HF**<br>BMI: 1.39 (1.26, 1.54)<br>WC: 1.49 (1.32, 1.68)<br>WHR: 1.57 (1.37, 1.80)<br>BSI: 1.25 (1.10, 1.43)<br>WWI 1.44 (1.27, 1.63)<br>BRI: 1.46 (1.32, 1.62)<br>RFM: 1.93 (1.60, 2.33)<br>**HFpEF**<br>BMI: 1.46 (1.24, 1.72)<br>WC: 1.56 (1.28, 1.90)<br>WHR: 1.48 (1.17, 1.86)<br>BSI: 1.20 (0.97, 1.48)<br>WWI: 1.38 (1.13, 1.69)<br>BRI: 1.48 (1.25, 1.75)<br>RFM: 2.04 (1.48, 2.81)<br>**HFrEF**<br>BMI: 1.34 (1.18, 1.52)<br>WC: 1.44 (1.24, 1.67)<br>WHR: 1.61 (1.36, 1.91)<br>BSI: 1.29 (1.10, 1.52)<br>WWI: 1.46 (1.25, 1.71)<br>BRI: 1.43 (1.26, 1.63)<br>RFM: 1.84 (1.46, 2.32)	8
45	Xing (2023)[55], UK, The UK Biobank,	2006-2010	483,316 participants, 55.4% women, 56.3 years	12.1 years (IQR 11.6-13.1 years)	Prevalent HF, prevalent cardiovascular<br>diseases, lack of bioimpedance analysis data, and loss<br>of follow-up	BMI, Arm fat index (AFI), Trunk fat index (TFI), leg fat index (LFI)	Incident HF	Electronic health record linkage	3134	-	Age, race, sex, BMI	J-shaped	HRs per SD higher adiposity measures	BMI: 1.67 (1.63-1.71)<br>AFI: 1.00 (0.96-1.05)<br>TFI: 1.00 (0.93-1.07)<br>LFI: 0.78 (0.72-0.84)	9

Table S5. Other subgroup analyses of BMI, waist circumference, and waist-hip ratio and incident heart failure.

Study characteristics	BMI, per 5 Kg/m^2 higher					Waist circumference per 10cm higher					Waist-hip ratio per 0.1unit higher				
	N	RR (95% CI)	I^2, %	P_{het}^*	P_{het}†	N	RR (95% CI)	I^2, %	P_{het}^*	P_{het}†	N	RR (95% CI)	I^2, %	P_{het}^*	P_{het}†
**All studies**	32	1.42 (1.40-1.44)	94.4	<0.001		13	1.28 (1.26-1.31)	75.8	<0.001		9	1.33 (1.28-1.37)	94.9	<0.001	
**Underweight excluded**															
Yes	9	1.39 (1.36-1.42)	87.3	<0.001	0.005	5	1.27 (1.22-1.32)	0.0	0.41	0.57	3	1.16 (1.09-1.23)	89.6	<0.001	<0.001
No	23	1.44 (1.42-1.47)	95.4	<0.001		9	1.29 (1.26-1.32)	83.8	<0.001		6	1.40 (1.35-1.46)	95.5	<0.001	
**Assessment of measures**															
Measured	27	1.43 (1.41-1.45	94.7	<0.001	0.45	12	1.28 (1.26-.131)	78.5	<0.001	0.60	7	1.42 (1.36-1.47)	94.8	<0.001	<0.001
Self-reported	5	1.40 (1.35-1.46)	92.9	<0.001		2	1.26 (1.20-1.34)	54.6	0.14		2	1.10 (1.03-1.17)	0.0	0.35	
**Events, n**															
< 500	16	1.31 (1.26-1.35)	80.3	<0.001	<0.001	8	1.27 (1.22-1.32)	40.7	0.11	0.67	3	1.22 (1.14-1.31)	89.4	0.001	<0.001
500-1000	6	1.38 (1.33-1.44)	46.0	0.10		3	1.27 (1.22-1.32)	72.7	0.03		3	1.17 (1.10-1.24)	89.1	<0.001	
>1000	10	1.46 (1.43-1.48)	97.9	<0.001		3	1.29 (1.26-1.33)	94.1	<0.001		3	1.54 (1.46-1.62)	97.1	<0.001	
**HF ascertainment**															
Adjudicated	13	1.35 (1.32-1.38)	88.0	<0.001	<0.001 /<0.001§	8	1.28 (1.25-1.31)	85.1	<0.001	0.41	4	1.46 (1.39-1.54)	96.8	<0.001	<0.001
Record linkage	17	1.45 (1.43-1.48)	95.6	<0.001		6	1.30 (1.25-1.36)	14.8	0.32		5	1.22 (1.17-1.28)	88.6	<0.001	
Self-reported	1	1.84 (1.69-2.01)	100.0	-		-	-	-	-		-	-	-	-	
**Study quality score**															
0-6	15	1.31 (1.28-1.35)	89.5	<0.001	<0.001	6	1.32 (1.29-1.36)	79.7	<0.001	<0.001	4	1.31 (1.26-1.37)	97.9	<0.001	0.41
7-9	17	1.46 (1.44-1.49)	95.6	<0.001		8	1.23 (1.20-1.27)	57.3	0.02		5	1.35 (1.28-1.42)	75.2	0.003	
**Effect size reported or estimated**															
Directly reported	18	1.46 (1.44-1.49)	96.2	<0.001	<0.001	11	1.28 (1.25-1.30)	79.8	<0.001	0.15	6	1.32 (1.27-1.37)	96.5	<0.001	0.65
Estimated	14	1.37 (1.34-1.40)	83.2	<0.001		3	1.35 (1.26-1.44)	0.0	0.38		3	1.34 (1.26-1.43)	87.0	<0.001	
**Adjustment for confounders**															
**Age**															-
Yes	28	1.42 (1.40-1.44)	95.0	<0.001	0.02	12	1.28 (1.25-1.30)	76.2	<0.001	0.01	9	1.33 (1.28-1.37)	94.9	<0.001	
No	4	1.57 (1.45-1.71)	0.0	0.61		2	1.49 (1.33-1.66)	0.0	0.44		0		-	-	
**Sex**															
Yes	19	1.48 (1.46-1.51)	93.9	<0.001	<0.001	8	1.28 (1.25-1.30)	83.5	<0.001	0.29	6	1.40 (1.35-1.46)	95.5	<0.001	<0.001
No	13	1.30 (1.27-1.33)	92.7	<0.001		6	1.31 (1.25-1.37)	49.9	0.08		3	1.16 (1.09-1.23)	89.6	<0.001	
**Ethnicity**															
Yes	6	1.49 (1.46-1.52)	98.6	<0.001	<0.001	3	1.28 (1.25-1.32)	94.1	<0.001	0.87	2	1.89 (1.75-2.04)	90.5	0.001	<0.001
No	26	1.38 (1.35-1.40)	85.1	<0.001		11	1.28 (1.24-1.32)	49.3	0.03		7	1.22 (1.18-1.27)	87.0	<0.001	
**Education**															
Yes	12	1.37 (1.34-1.39)	81.9	<0.001	<0.001	8	1.29 (1.26-1.32)	82.5	<0.001	0.22	6	1.37 (1.32-1.43)	96.3	<0.001	0.002
No	20	1.47 (1.44-1.49)	95.9	<0.001		6	1.25 (1.20-1.31)	58.6	0.03		3	1.22 (1.15-1.30)	81.6	0.004	
**Smoking**															
Yes	22	1.33 (1.31-1.35)	90.7	<0.001	<0.001	11	1.28 (1.25-1.31)	78.3	<0.001	0.65	8	1.32 (1.27-1.37)	95.5	<0.001	0.40
No	10	1.62 (1.59-1.66)	92.5	<0.001		3	1.30 (1.23-1.37)	72.7	0.03		1	1.38 (1.25-1.52)	0.0	-	
**Alcohol**															
Yes	11	1.39 (1.36-1.41)	90.2	<0.001	<0.001	7	1.29 (1.26-1.32)	84.6	<0.001	0.28	6	1.37 (1.32-1.43)	96.3	<0.001	0.002
No	21	1.45 (1.43-1.48)	95.4	<0.001		7	1.26 (1.22-1.31)	55.5	0.04		3	1.22 (1.15-1.30)	0.0	0.004	
**Physical activity**															

Yes	12	1.36 (1.33-1.39)	84.8	<0.001	<0.001	7	1.24 (1.20-1.28)	68.4	0.004	0.002	6	1.22 (1.17-1.28)	85.8	<0.001	<0.001
No	20	1.47 (1.45-1.50)	95.7	<0.001		7	1.32 (1.28-1.35)	76.4	<0.001		3	1.47 (1.40-1.55)	97.8	<0.001	
**Adjustment for potential mediators**															
**Hypertension**															
Yes	9	1.21 (1.17-1.24)	89.9	<0.001	<0.001	1	1.38 (1.10-1.72)	0.0	-	0.53	0	-	-	-	-
No	23	1.48 (1.46-1.50)	93.2	<0.001		13	1.28 (1.26-1.31)	77.5	<0.001		8	1.33 (1.28-1.37)	94.9	<0.001	
**Blood pressure**															
Yes	5	1.33 (1.28-1.37)	89.9	<0.001	<0.001	2	1.38 (1.28-1.49)	0.0	0.89	0.05	2	1.67 (1.47-1.89)	0.0	0.93	<0.001
No	27	1.44 (1.42-1.46)	94.7	<0.001		12	1.28 (1.25-1.30)	77.5	<0.001		7	1.30 (1.26-1.35)	95.8	<0.001	
Diabetes															
Yes	14	1.26 91.23-1.29)	87.6	<0.001	<0.001	6	1.27 (1.21-1.33)	44.9	0.11	0.59	3	1.26 (1.18-1.36)	92.8	<0.001	0.13
No	18	1.49 (1.47-1.52)	94.5	<0.001		8	1.29 (1.26-1.31)	84.2	<0.001		6	1.35 (1.30-1.40)	96.1	<0.001	
**Ischaemic heart disease**															
Yes	10	1.30 (1.27-1.34)<br>1.46 (1.44-1.48)	87.8	<0.001	<0.001	2	1.37 (1.23-1.53)	0.0	0.96	0.21	1	1.66 (1.39-1.98)	95.4	-	0.01
No	22		95.0	<0.001		12	1.28 (1.25-1.30)	78.9	<0.001		8	1.32 (1.27-1.36)	100.0	<0.001	
**Atrial fibrillation**															
Yes	3	1.24 (1.18-1.29)	91.4	<0.001	0.001	1	1.17 (1.12-1.22	100	-	<0.001	0	-	-	-	-
No	29	1.44 (1.42-1.46)	94.2	<0.001		13	1.31 (1.28-1.34)	62.5	0.001		9	1.33 (1.28-1.37)	94.9	<0.001	
**Valvular heart disease**															
Yes	3	1.33 (1.29-1.38)	84.2	0.002	<0.001	2	1.19 (1.14-1.24)	82.5	0.02	<0.001	1	1.66 (1.39-1.98)	100.0	-	0.01
No	29	1.44 (1.42-1.46)	94.6	<0.001		12	1.31 (1.28-1.34)	65.1	0.001		8	1.32 (1.27-1.36)	95.4	<0.001	
**Left ventricular hypertrophy**															
Yes															
No	4	1.19 (1.11-1.26)	78.8	<0.001	<0.001	2	1.30 (1.15-1.48)	0.0	0.55	0.80	0	-	-	-	-
	28	1.44 (1.42-1.45)	94.6	0.003		12	1.28 (1.26-1.31)	79.3	<0.001		9	1.33 (1.28-1.37)	94.9	<0.001	
**Cholesterol**															
Yes	9	1.27 (1.23-1.30)	91.4	<0.001	<0.001	4	1.24 (1.17-1.30)	40.3	0.17	0.14	2	1.20 (1.11-1.29)	93.8	<0.001	0.004
No	23	1.47 (1.45-1.50)	93.9	<0.001		10	1.29 (1.26-1.32)	80.6	<0.001		7	1.36 (1.31-1.41)	95.5	<0.001	
**Lipid lowering drugs**															
Yes	3	1.14 (1.09-1.18)	89.4	<0.001	<0.001	1	1.39 (1.26-1.53)	0.0	-	0.12	1	1.68 (1.41-2.00)	100.0	-	0.008
No	29	1.46 (1.44-1.48)	92.7	<0.001		13	1.28 (1.25-1.30)	76.5	<0.001		8	1.32 (1.27-1.36)	95.3	<0.001	
**Adjustment for key intermediate factors**[‡]															
Yes	7	1.30 (1.26-1.34)	89.7	<0.001	<0.001	2	1.37 (1.23-1.53)	0.0	0.96	0.21	1	1.66 (1.39-1.98)	100.0	-	0.01
No	25	1.45 (1.43-1.47)	94.7	<0.001		12	1.28 (1.25-1.30)	78.9	<0..001		8	1.32 (1.27-1.36)	95.4	<0.001	

N= number of studies in subgroup meta-analysis (this is not always equal to the total number of studies in the overall analysis). BMI indicates body mass index; CI, confidence interval; and RR, relative risk.

*P for heterogeneity within each subgroup.

†P for heterogeneity between subgroups.

‡Adjustment for key intermediate factors (BP/hypertension, diabetes and ischaemic heart disease)

§P for heterogeneity between adjudicated and record linkage (excluding self-reported HF events)

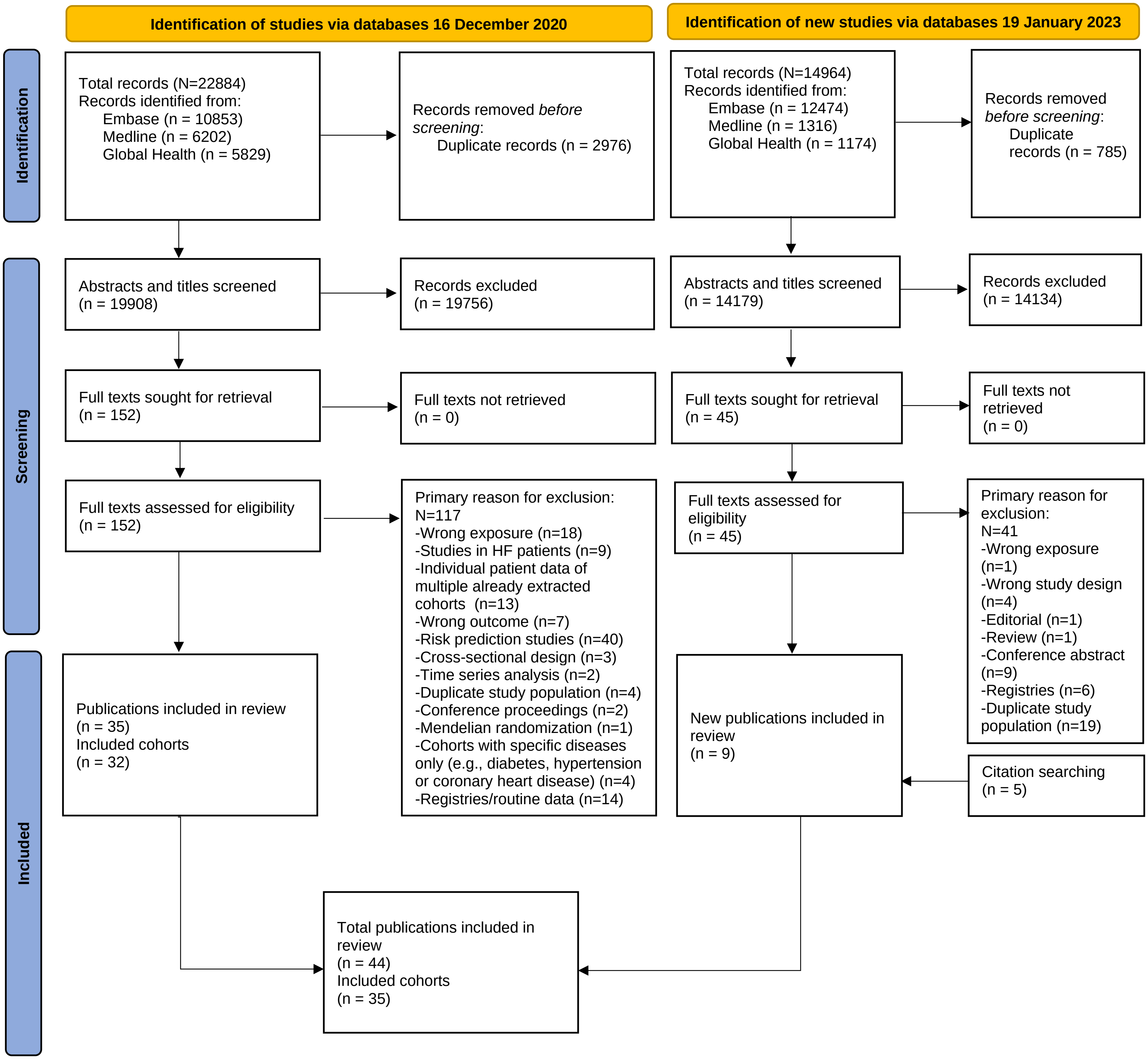

Figure S1: PRISMA flowchart of study selection

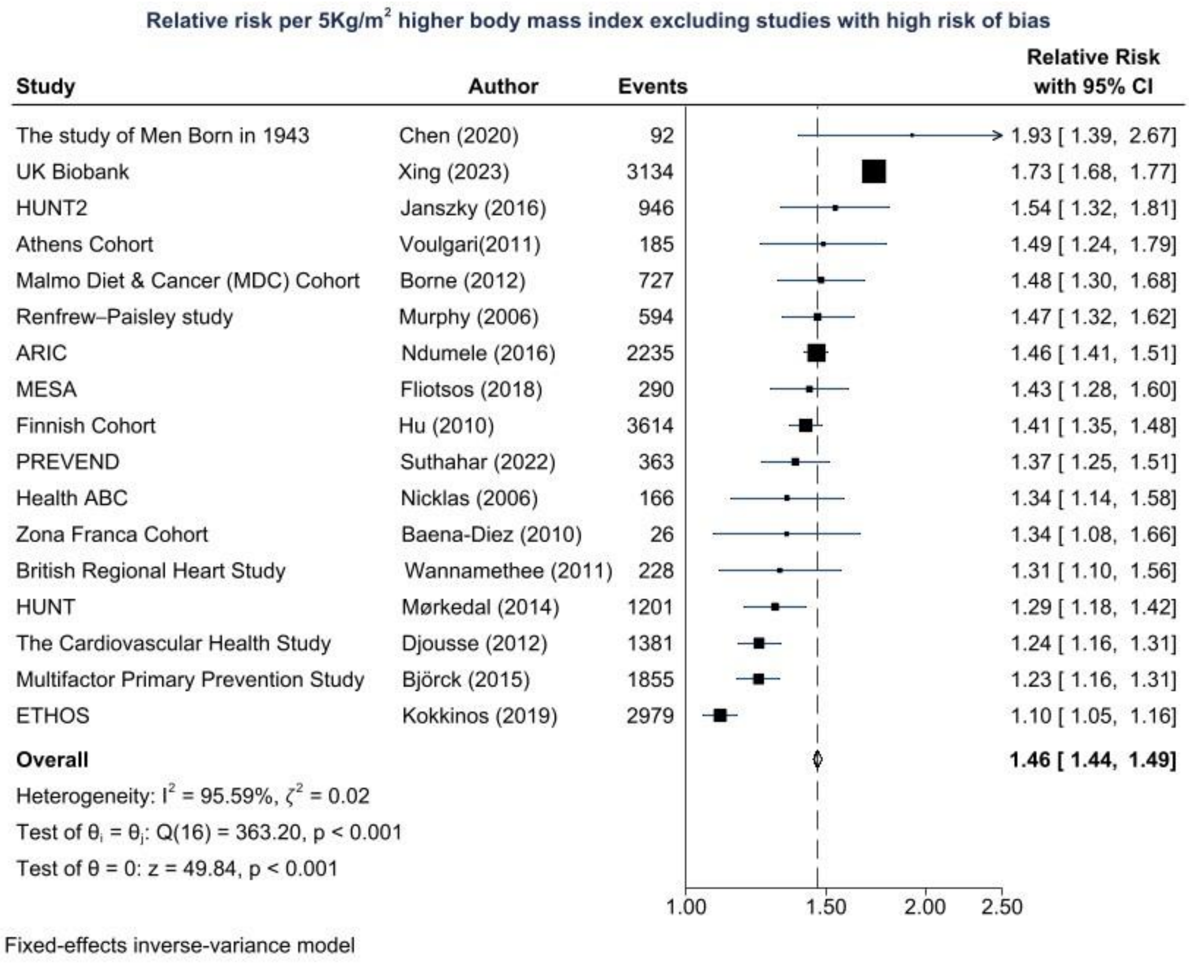

Figure S2: Dose-response meta-analysis of BMI and HF incidence excluding studies with high risk of bias

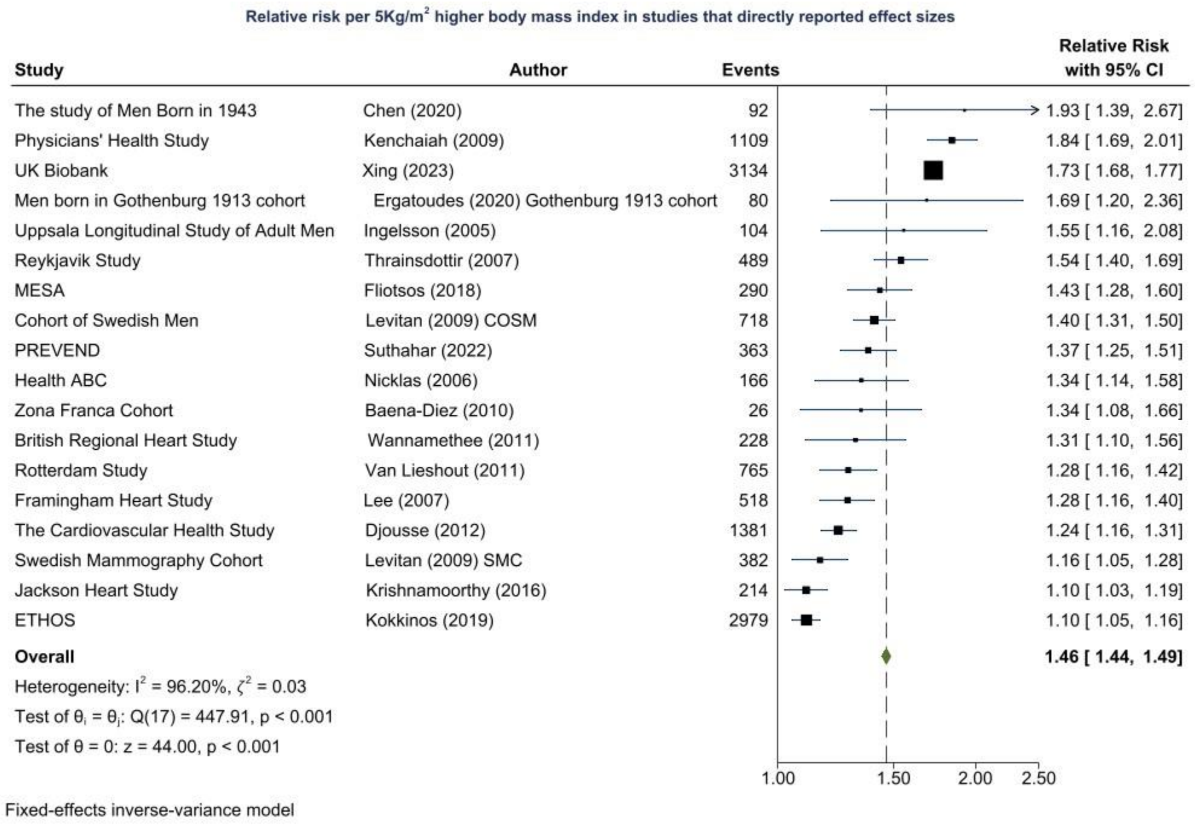

Figure S3: Dose-response meta-analysis of BMI and HF incidence in studies that directly reported effect sizes

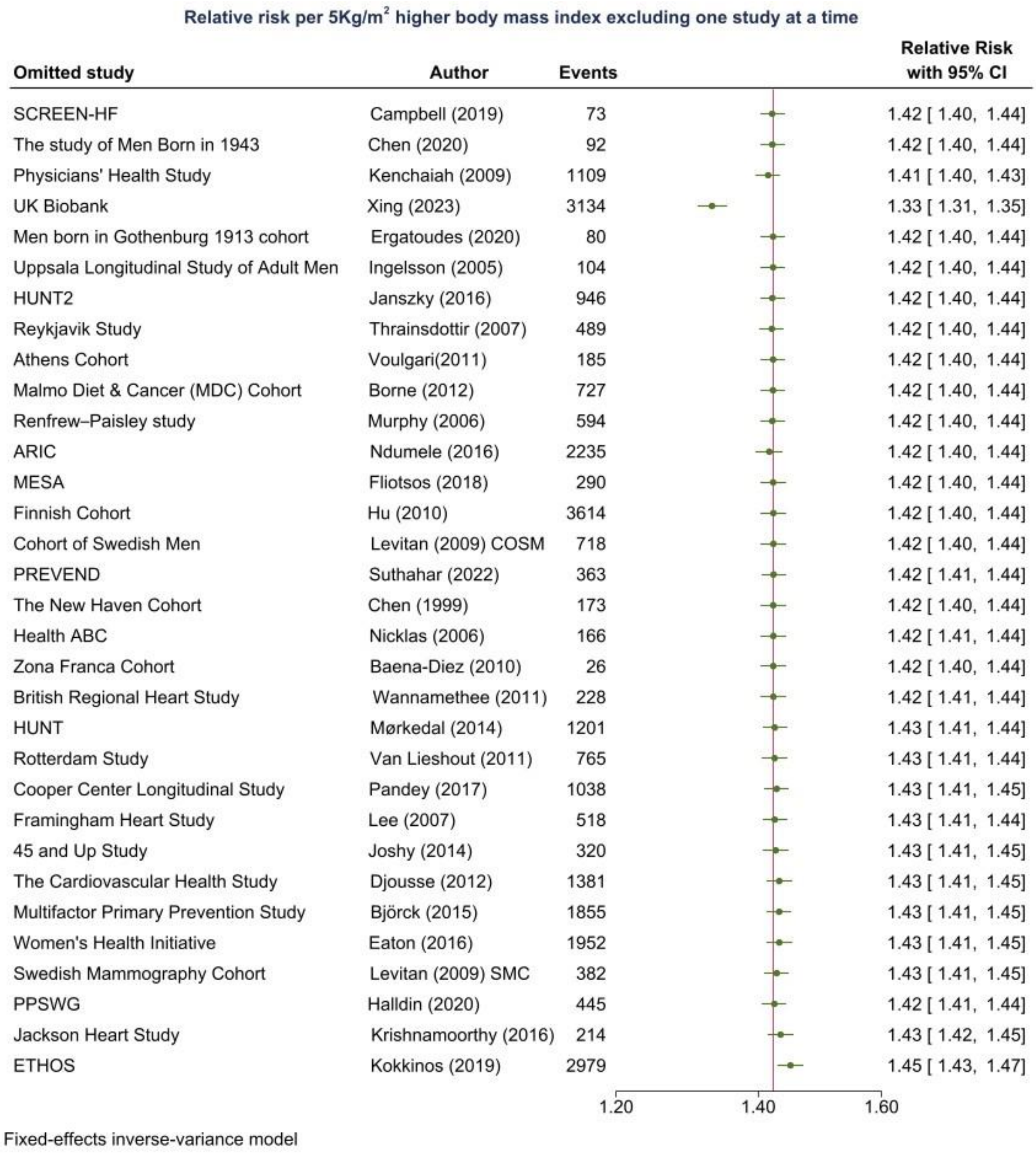

Figure S4: Meta-analysis of BMI and HF risk excluding one study at a time

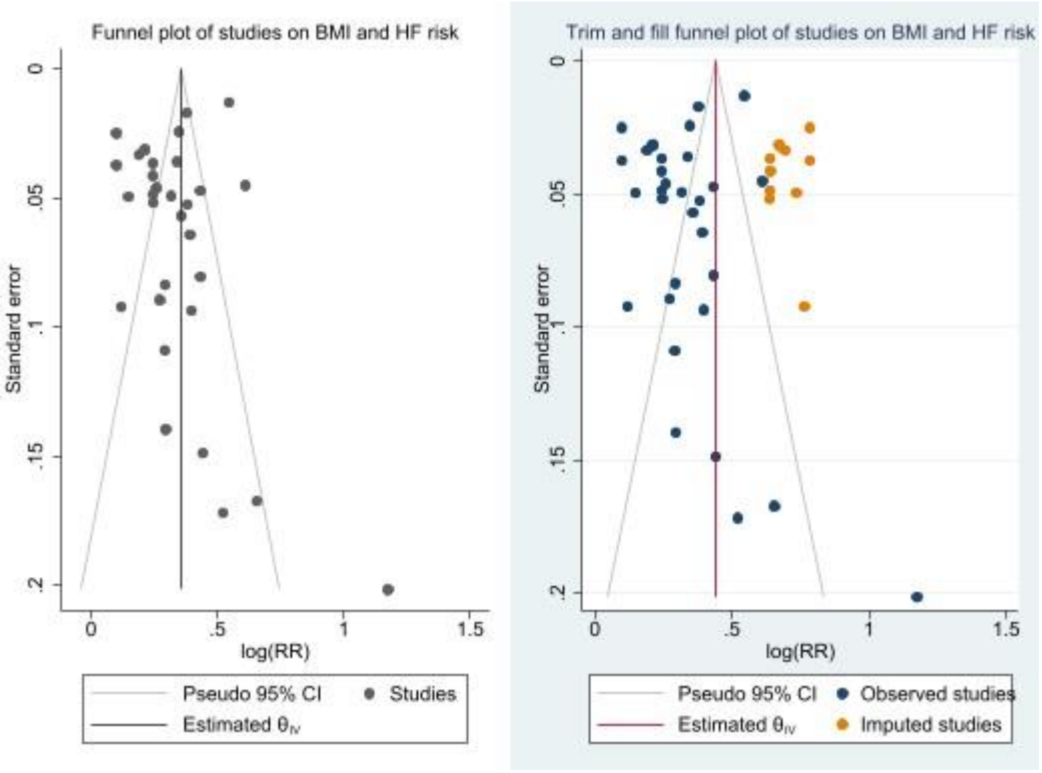

Figure S5: Funnel plot and Trim and fill plot of studies on BMI and HF risk

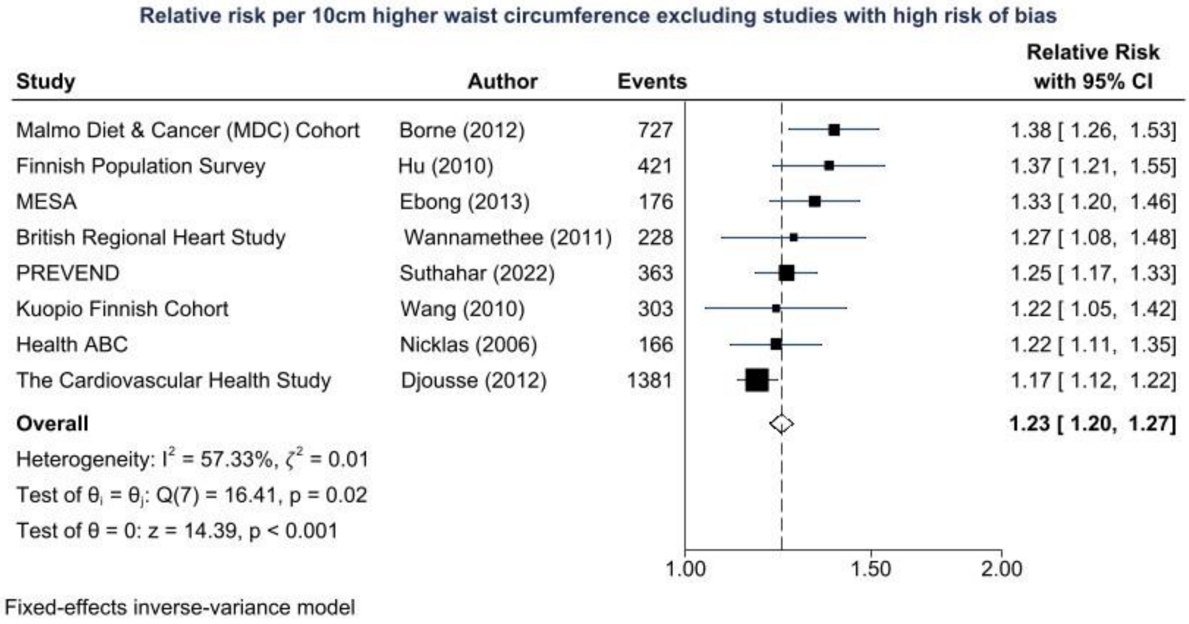

Figure S6: Dose-response meta-analysis of waist circumference and HF incidence excluding studies with high risk of bias

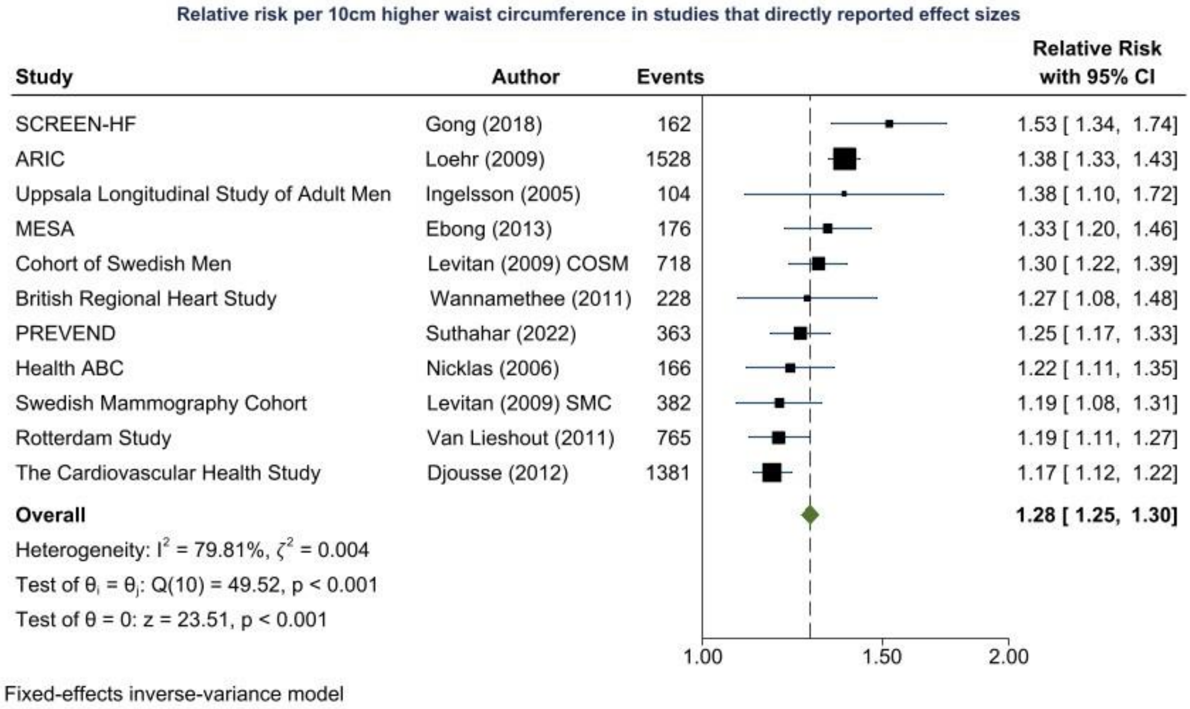

Figure S7: Dose-response meta-analysis of waist circumference and HF incidence in studies that directly reported effect sizes

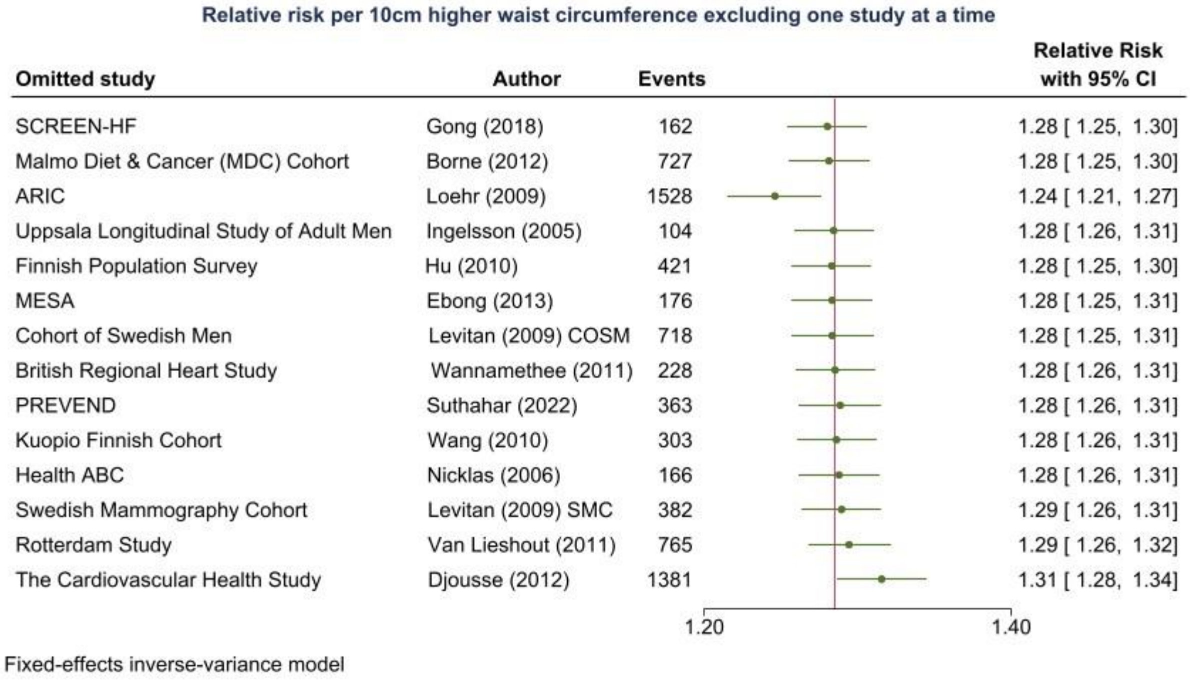

Figure S8: Meta-analysis of waist circumference and HF risk excluding one study at a time

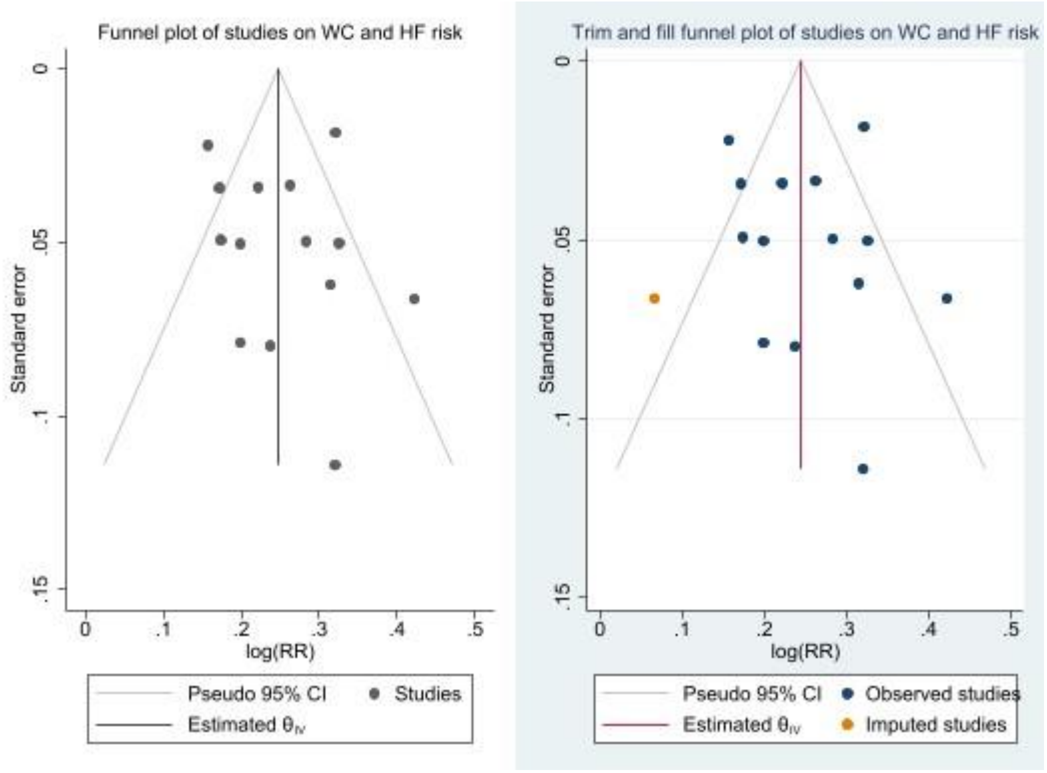

Figure S9: Funnel plot and Trim and fill plot of studies on waist circumference and HF risk

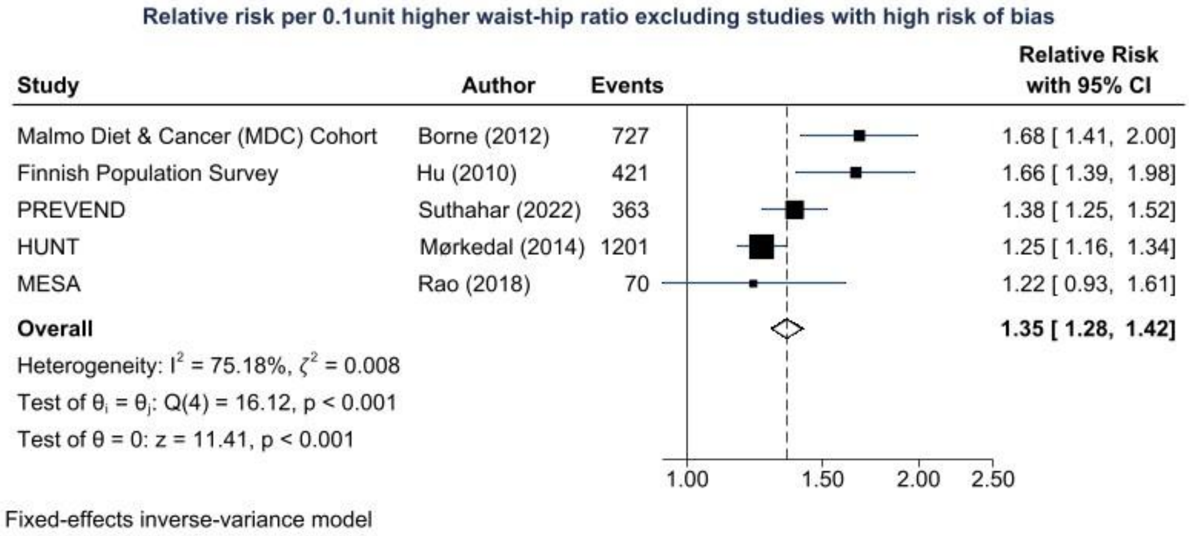

Figure S10: Dose-response meta-analysis of waist-hip ratio and HF excluding studies with high risk of bias

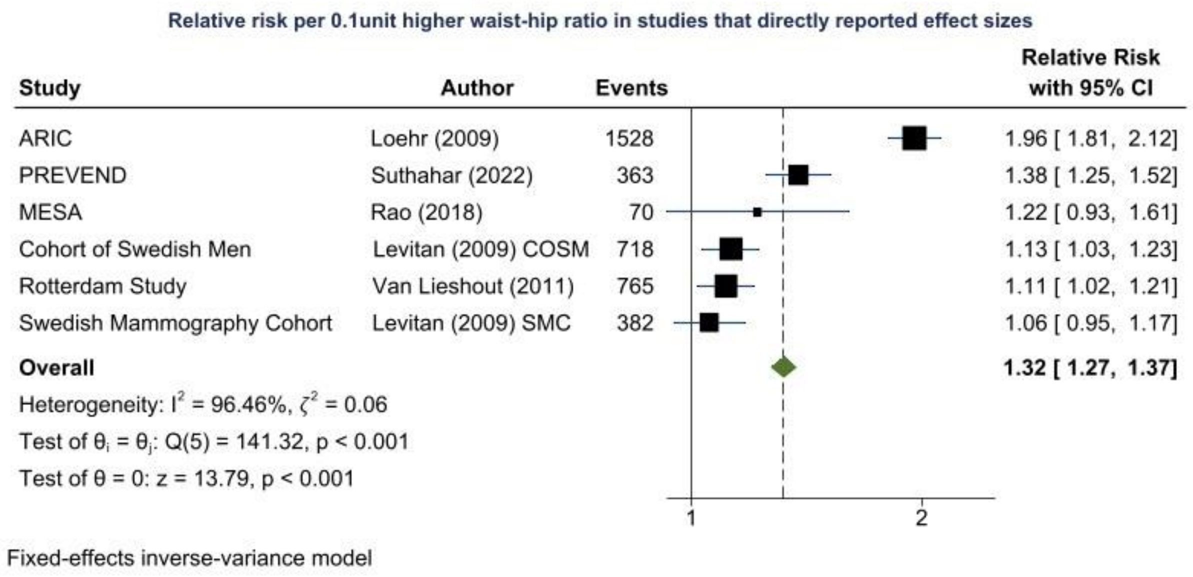

Figure S11: Dose-response meta-analysis of waist-hip ratio and HF incidence in studies that directly reported effect sizes

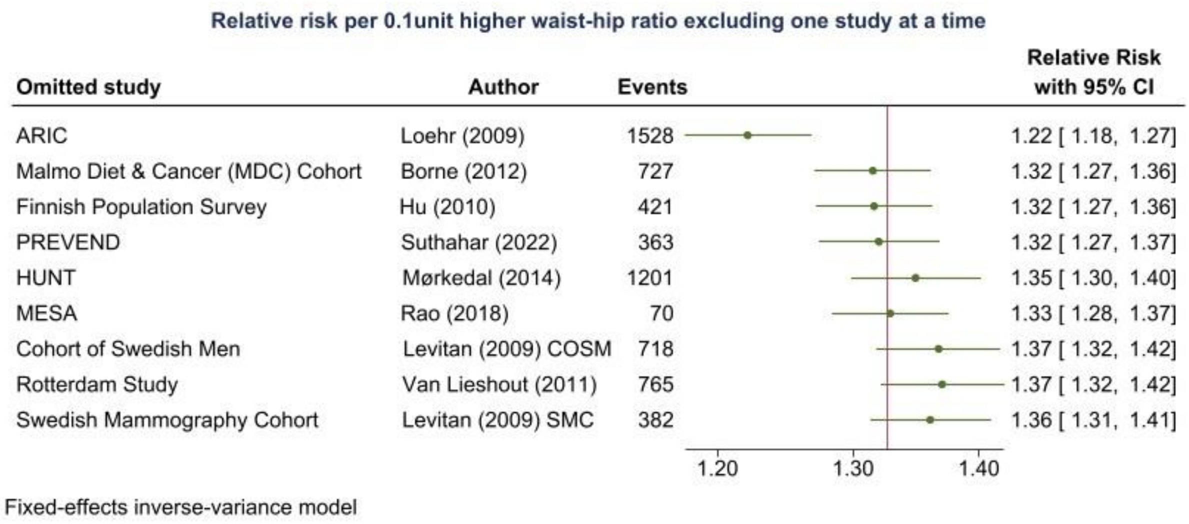

Figure S12: Meta-analysis of waist-hip ratio and HF risk excluding one study at a time

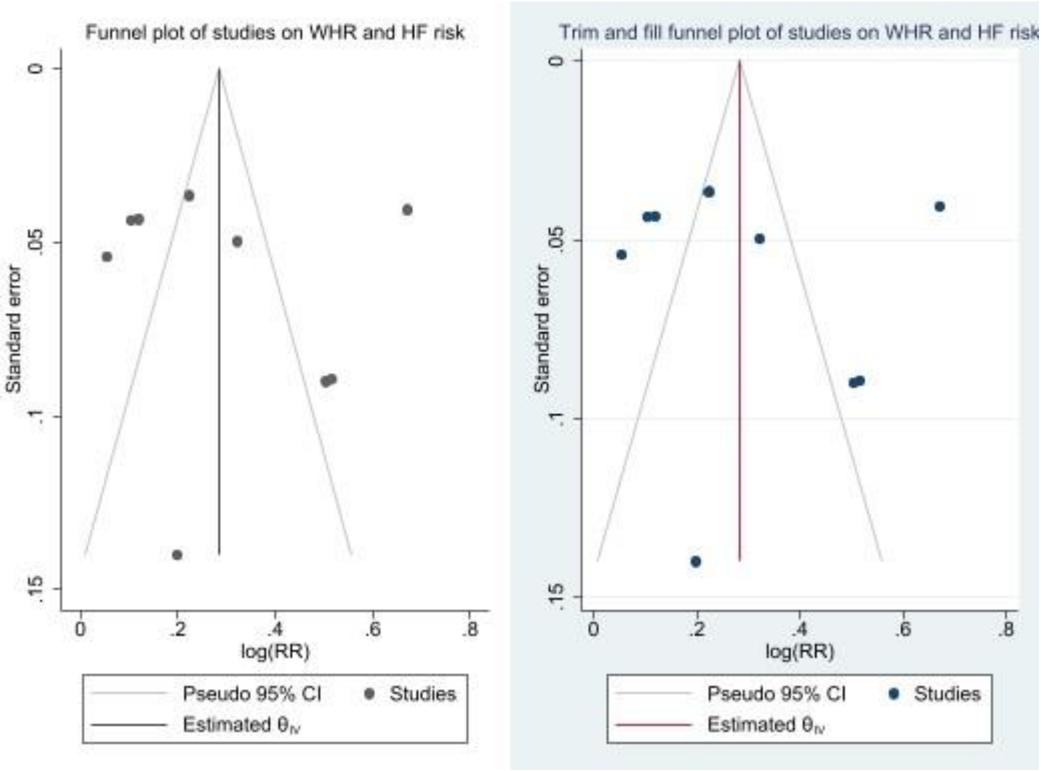

Figure S13: Funnel plot and Trim and fill plot of studies on waist-hip ratio and HF risk
